# New insights into the role of cyanide in the promotion of seed germination in tomato

**DOI:** 10.1186/s12870-021-03405-8

**Published:** 2022-01-11

**Authors:** Lu-Lu Yu, Cui-Jiao Liu, Ye Peng, Zheng-Quan He, Fei Xu

**Affiliations:** 1 Applied Biotechnology Center, Wuhan University of Bioengineering, Wuhan, 430415 China; 2 Biotechnology Research Center, China Three Gorges University, Yichang, 443002 China

**Keywords:** Cyanide, Respiration, Seed germination, Tomato

## Abstract

**Background:**

Cyanide is a natural metabolite that exists widely in plants, and it is speculated to be involved in the regulation of various growth and development processes of plants in addition to being regarded as toxic waste. Previous studies have shown that exogenous cyanide treatment helps to improve seed germination, but the mechanism is still unclear. In this study, tomato (*Solanum lycopersicum* cv. Alisa Craig) was used as the material, and the effects of cyanide pretreatment at different concentrations on tomato seed germination were investigated.

**Results:**

The results showed that exogenous application of a lower concentration of cyanide (10 μmol/L KCN) for 12 h strongly increased the tomato seed germination rate. RNA-Seq showed that compared with the control, a total of 15,418 differentially expressed genes (*P*<0.05) were obtained after pretreatment with KCN for 12 h, and in the next 12 h, a total of 13,425 differentially expressed genes (*P*<0.05) were regulated. GO and KEGG analyses demonstrated that exogenous KCN pretreatment was involved in regulating the expression (mainly downregulation) of seed storage proteins, thereby accelerating the degradation of stored proteins for seed germination. In addition, KCN pretreatment was also involved in stimulating glycolysis, the TCA cycle and oxidative phosphorylation. Notably, it is shown that KCN acted on the regulation of plant hormone biosynthesis and perception, i.e., down-regulated the gene expression of ABA biosynthesis and signal transduction, but up-regulated the expression of genes related to GA biosynthesis and response. Consistent with this, plant hormone measurements confirmed that the levels of ABA were reduced, but GA levels were induced after pretreatment with KCN.

**Conclusion:**

These findings provide new insights into the regulation of seed germination by cyanide, that is cyanide-mediated seed germination occurs in a time- and dose-dependent manner, and is related to the mobilization of energy metabolism and the regulation of some plant hormone signals.

**Supplementary Information:**

The online version contains supplementary material available at 10.1186/s12870-021-03405-8.

## Background

Seed germination is a crucial stage for plant development and agricultural production, which is a complex process involving various physical and biochemical cues, determined by exogenous (e.g. temperature, water, oxygen, light) and endogenous (phytohormones) factors [1]. The physical, physiological and biochemical processes of seed germination have been described in detail for several species [2]. In the process of seed germination, water is absorbed by the embryo, resulting in the rehydration and expansion of the cells. In general, the imbibition of water by seeds before germination can be divided into three phases: phase I, which is denoted by a rapid initial water uptake (imbibition), where a rapid activation of respiration and protein synthesis (utilizing stored mRNAs already present in the seed) is observed; phase II, which is marked by a plateau in water uptake, where massive degradation of food reserves (storage proteins, fats and carbohydrates) occurs; and phase III, which comprises a further increase in water uptake at the end of germination, as the embryonic axis elongates [2, 3].

It is widely accepted that hormones are involved in the regulation of seed dormancy and germination [4, 5]. Abscisic acid (ABA) is an essential repressor of seed germination, while gibberellins (GAs) are a promoter of seed germination, and it is apparent that ABA and GAs antagonistically regulate seed germination [6, 7]. During seed germination, GA increases the growth potential of the embryo and is necessary to overcome the mechanical restraint conferred by the seed-covering layers by weakening the tissues surrounding the radicle [7]. Conversely, ABA can prevent the weakening of the cell wall induced by GA [4]. It has been demonstrated that ABA-insensitive factors, including ABI3 and ABI5, are involved in the promotion of seed dormancy and inhibition of seed germination [8, 9]. Moreover, recent studies have pointed out that DELAY OF GERMINATION-1 (DOG1), which is a master regulator of primary dormancy (PD), acts in concert with ABA to delay germination [10, 11]. Likewise, GA- biosynthetic and signaling mutants have strong seed dormancy phenotypes in many plant species and are unable to germinate unless the seed coat and endosperm are removed [5]. In the GA signaling pathway, evidences have shown that DELLA proteins (negative regulators of the GA signaling transduction pathway) are absolutely required for seed dormancy. For instance, the accumulation of DELLAs in seeds can enhance ABA-mediated seed dormancy; in contrast, DELLA mutant seeds can germinate even with low GA levels [12, 13]. In addition, detailed studies have revealed that the gene expression of DELLAs is promoted by exogenous ABA [12] and inhibited by GA [14]. Thus, the involvement of the hormonal balance and interaction between ABA and GA in the regulation of seed germination and dormancy is critical and complex.

In addition to GAs and ABA, other hormones such as ethylene, auxins, brassinosteroids (BRs), and jasmonates (JAs), also play a role in the control of seed germination [1, 7, 15]. More interestingly, some small molecules such as nitric oxide (NO), reactive oxygen species (ROS) and cyanide (e.g. hydrogen cyanide, HCN; potassium cyanide, KCN) were proposed to play important roles in the regulation of seed germination [2, 16]. However, the role of these small molecules in the process of seed germination regulation is not clear and remains controversial. With respect to cyanide, it has been shown that seed germination is promoted by cyanide (HCN/KCN) in a dose-dependent manner [17]. Cyanide causes lethal toxicity to animals by binding to and inactivating cytochrome *c* oxidase in the mitochondria [17]. In contrast, due to the existence of a cyanide-insensitive pathway mediated by alternative oxidase (AOX) in the electron transport chain of plant mitochondria, the toxic effect of cyanide on cells is reduced [18]. Interestingly, in plants, cyanide is liberated from cyanogenic compounds (hydrolyzed in vacuoles) when plants are attacked by predators [19, 20] and is also a co-product of ethylene biosynthesis, where it is produced in stoichiometrically equal amounts to ethylene [17, 21]. Importantly, previous evidence supports that cyanide plays a dual role in plants; that is, it has a toxic effect at high concentrations and acts as a signal molecule at low concentrations [17, 22, 23]. This dual effect might depend on the concentration of cyanide, as well as on the status of a plant and its growth conditions [17].

In some plant species, cyanide at millimolar concentrations stimulated seed germination, but the stimulatory effect of cyanide was observed only when it has been subsequently eliminated from germination medium [16, 17, 24]. Moreover, it has been suggested that NO and ROS are involved in cyanide-mediated seed dormancy removal [16, 25]. The promotion effect of cyanide on germination has also been confirmed to interact with ethylene biosynthesis and signal transduction pathways in apple and sunflower seeds [26, 27]. However, the molecular mechanism of cyanide-mediated seed germination remains largely unknown and needs to be further studied. For example, is cyanide involved in the regulation of synthesis and signal transduction of hormones such as ABA and GA? Secondly, it is unknown whether cyanide acts as a signaling molecule affects the expression of germination-related genes, especially when it is at lower concentrations.

Therefore, the aim of the present study was to further reveal the molecular mechanism of cyanide-promoted seed germination. Cyanide was used in micromolar concentrations and the tomato seed germination was recorded. Moreover, combined with physiological and transcriptome analysis, the effects of cyanide on the biosynthesis and perception of plant hormones and substance metabolism were investigated and discussed.

## Results

### Cyanide pretreatment promotes tomato seed germination at lower concentrations

**Fig. 1:**
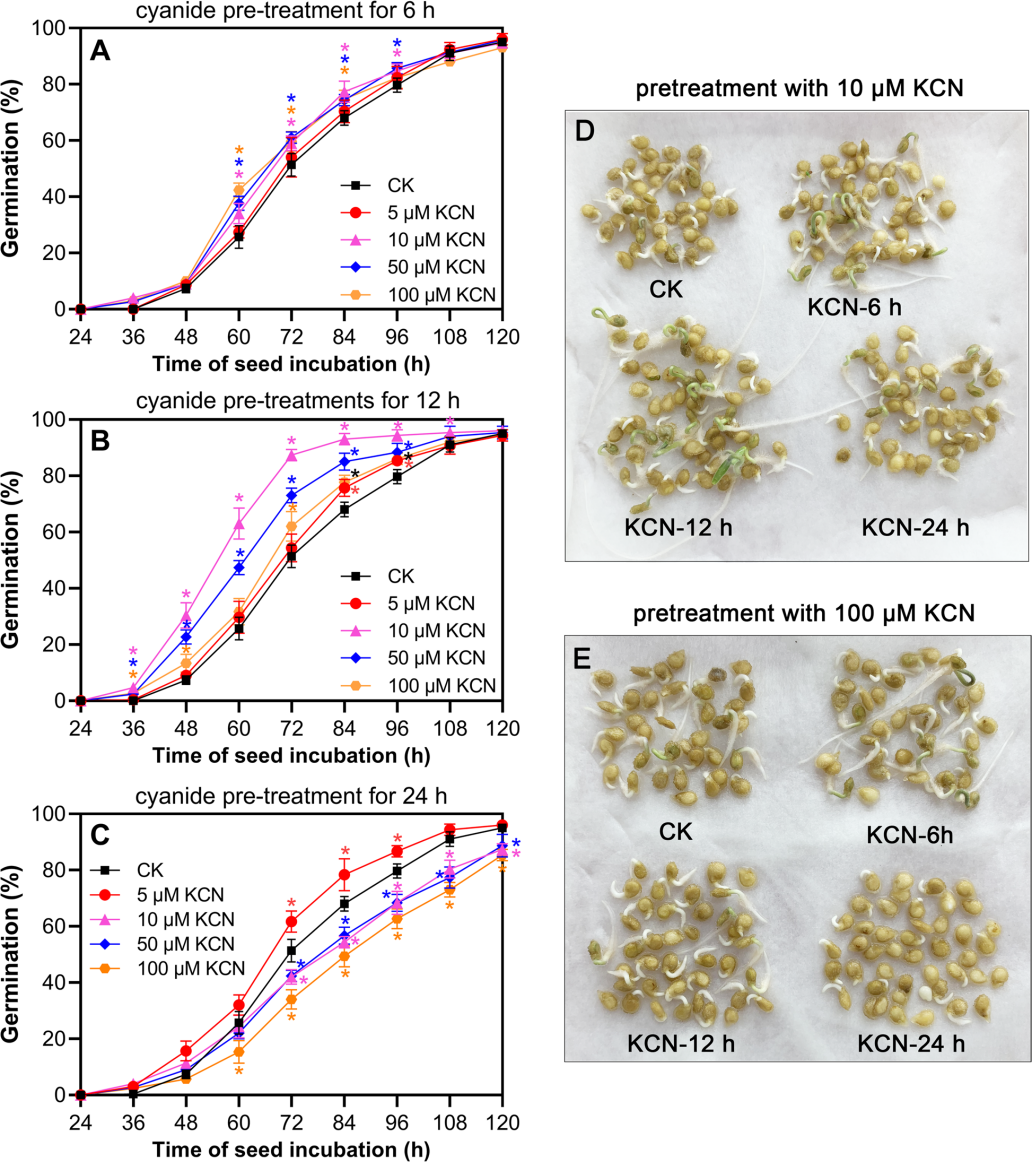
Effects of cyanide pretreatments on tomato seed germination. **A**-**C** The seed germination was recorded and compared after pretreatment with different concentrations of cyanide for 6 h (**A**), 12 h (**B**), and 24 h (**C**). Data are the means ±SD of three replicates from three independent experiments. Different colored asterisks represent the significant differences (one-way ANOVA, *P < 0.05) between cyanide-pretreated samples (KCN) and the control (CK). **D** and **E** After incubation with 10 μM KCN and 100 μM KCN for 6 h, 12 h, and 24 h, the representative seed germination morphology at 72 h are shown

In this study, different concentrations of cyanide were used to investigate the effects of exogenous cyanide pretreatment on tomato seed germination. As shown in Fig. 1, tomato germination was promoted by lower concentrations of cyanide, such as 10 μM and 50 μM KCN, while a higher concentration of cyanide pretreatment, such as 100 μM KCN, inhibited seed germination (Fig. 1). However, it should be noted that the incubation time of cyanide also affected the germination of tomato seeds. The results showed that pretreatment with 10 μM and 50 μM KCN for 6 h and 12 h promoted seed germination but inhibited seed germination when the incubation time was extended to 24 h (Fig. 1A-C). In addition, although there was no significant difference in seed germination after pretreatment with 5 μM KCN for 6 h and 12 h, better seed germination was observed after 24 h of incubation compared to the control (Fig. 1A-C). In contrast, pretreatment with 100 μM KCN for a short time, such as 6 h, showed a little promotion effect on tomato seed germination, although long-term incubation (e.g. 12 h and 24 h) was detrimental to germination (Fig. 1). In comparison, the results showed that pretreatment with 10 μM KCN for 12 h was the optimal time for the promotion of tomato seed germination (Fig. 1D).

### Cyanide pretreatment stimulates tomato seed respiration

**Fig. 2:**
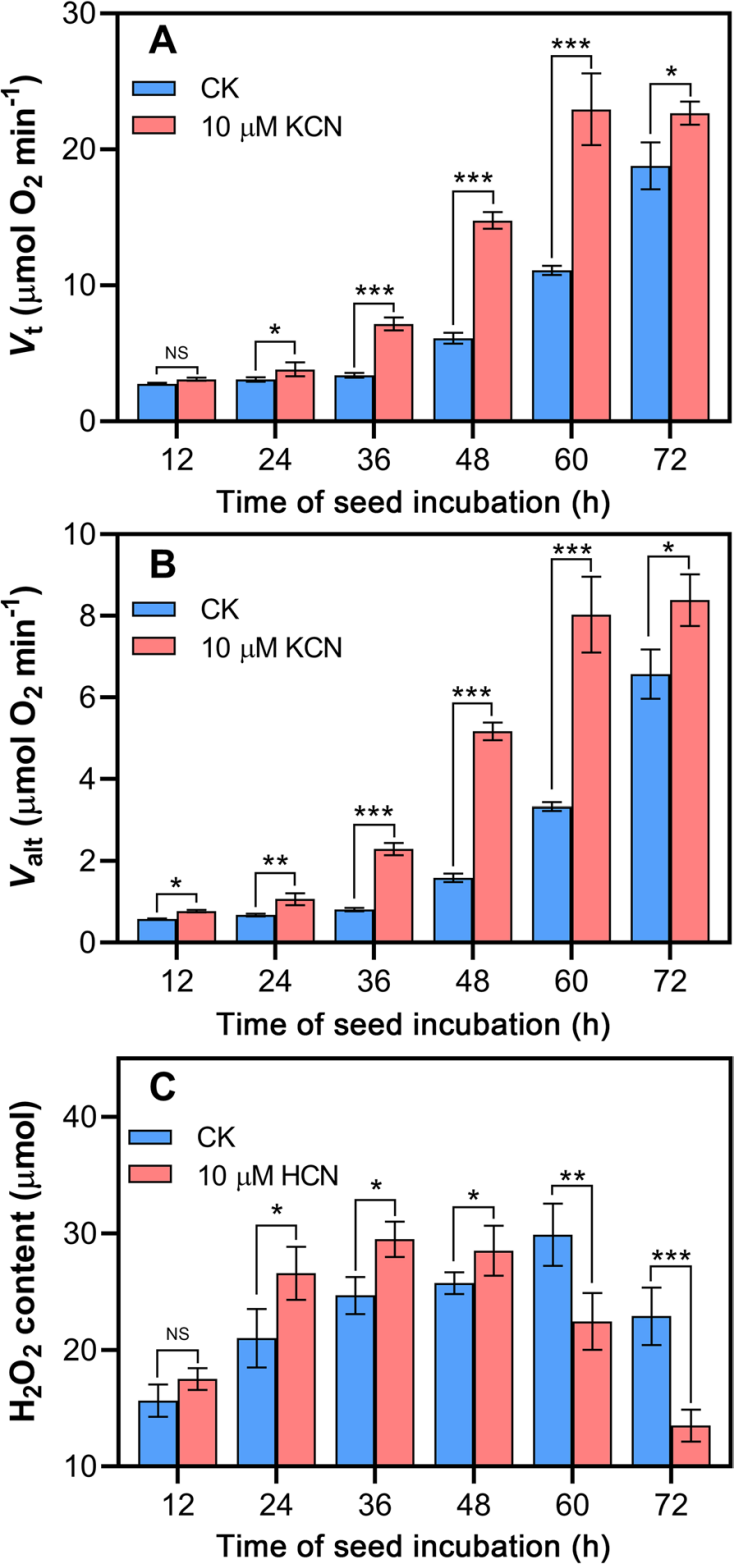
Changes in respiration and H_2_O_2_ content after 10 μM KCN pretreatment. Comparison of the rates of *V*_t_ (**A**) and *V*_alt_ (**B**), and the H_2_O_2_ content (**C**) with or without 10 μM KCN pretreatment for 12 h. *V*_t_, total respiration. *V*_alt_, alternative oxidase pathway respiration. In each test, 10 seeds were used to measure respiration or H_2_O_2_ production. Data are the means ±SD of three replicates from three independent experiments. Asterisks represent the significant differences (one-way ANOVA, *P < 0.05, **P < 0.01, and ***P < 0.001) between cyanide-pretreated samples (KCN) and the control (CK)

Given that 10 μM KCN pretreatment for 12 h was the best condition for tomato seed germination, we next investigated the effects of 10 μM KCN pretreatment on the seed respiration rate. The results showed that the total respiration (*V*_t_) rate was induced by cyanide pretreatment for 12 h (Fig. 2A). Moreover, in the subsequent germination process, the *V*_t_ of the seeds pretreated with cyanide was much higher than that of the control seeds (Fig. 2A). After 36 h of incubation, there was approximately a 2-fold higher rate of *V*_t_ observed in the cyanide-pretreated seeds when compared to the control.

Similar to changes in *V*_t_, the alternative oxidase (AOX) pathway respiration (*V*_alt_) was also induced by 10 μM of KCN pretreatment, and a higher rate of *V*_alt_ was maintained in cyanide-pretreated seeds in the following process of germination when compared with the control (Fig. 2B). However, it was more prominent during the period of 24 h to 60 h if the differences in *V*_alt_ were compared between the cyanide-pretreated seeds and the control (Fig. 2B). These findings indicate that cyanide-promoted tomato seed germination was associated with enhanced total respiration and cyanide-resistant respiration.

It was proposed that cyanide is toxic to cytochrome *c* pathway respiration and induces ROS production in mitochondria [17]. Therefore, changes in hydrogen peroxide (H_2_O_2_) during tomato seed germination with or without 10 μM KCN pretreatment were assessed. As shown in Fig. 2C, cyanide pretreatment did not significantly induce H_2_O_2_ production in the early stage (12 h), but a higher level of H_2_O_2_ was observed in the following germination period (24 h to 48 h) compared to that of the control. After 60 h, it should be noted that the H_2_O_2_ level in the KCN-pretreated seeds was significantly reduced compared to the control (Fig. 2C). However, there was no significant difference in the maximum H_2_O_2_ content between cyanide-pretreated seeds and the control seeds during the germination process (Fig. 2C). Together, these findings indicate that cyanide pretreatment accelerated the production of ROS instead of its excessive accumulation.

### Transcriptome analysis of the effect of cyanide treatment on tomato seed germination

**Fig. 3:**
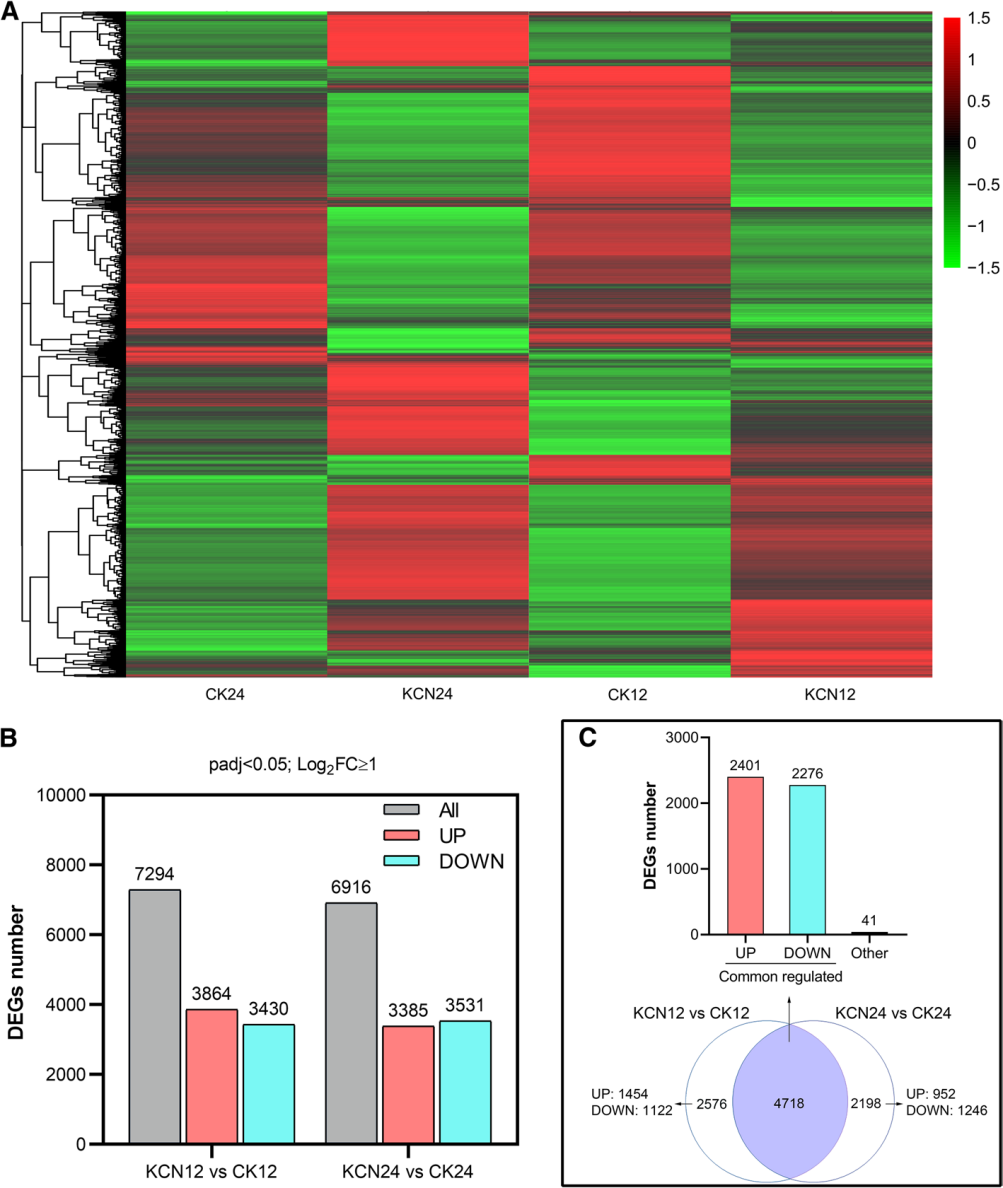
Expression profile of samples with or without cyanide pretreatment. **A** Hierarchical clustering analysis of normalized count data z-scores exhibited by DEGs of each sample at each time point. **B** The number of DEGs (padj< 0.05; log_2_FC ≥ 1) includes up-regulated and down-regulated genes in response to cyanide. **C** Venn diagram showing the common-regulated DEGs in KCN12 vs. CK12 and KCN24 vs. CK24; UP, up-regulated DEGs; DOWN, down-regulated DEGs; Other, DEGs are not consistently up-regulated or down-regulated in KCN12 vs. CK12 and KCN24 vs. CK24. DEGs, differentially expressed genes. FC, fold change

To further investigate the effects of cyanide pretreatment on the gene expression of tomato seeds, RNA-SEQ was carried out to reveal the possible mechanism of cyanide-promoted seed germination. In this study, seeds were pretreated with 10 μM KCN for 12 h (labeled KCN-12), and the remaining seeds were incubated under normal conditions for an additional 12 h (labeled KCN-24). Both sets of seeds were used for transcriptome analysis. The results showed that a large number of genes were regulated by cyanide (Fig. 3; Supplementary Fig. S1). A total of 15,418 and 13,425 differentially expressed genes (DEGs) were observed in KCN12 vs. CK12 and KCN24 vs. CK24, respectively. Of these, a total of 7294 and 6916 DEGs (log_2_FC ≥ 1) were significantly regulated by cyanide (Fig. 3B). Moreover, it should be noted that 4718 DEGs (log_2_FC ≥ 1) were identified in both KCN12 vs. CK12 and KCN24 vs. CK24, of which 2401 DEGs were jointly up-regulated and 2276 DEGs were jointly down-regulated (Fig. 3C). To confirm the reliability of the transcriptome sequencing, some DEGs were investigated by qRT-PCR. As shown in Supplementary Fig. S2, the results of qRT-PCR were generally consistent with the transcriptome data, suggesting a strong positive correlation between the qRT-PCR and transcriptome data.

**Fig. 4:**
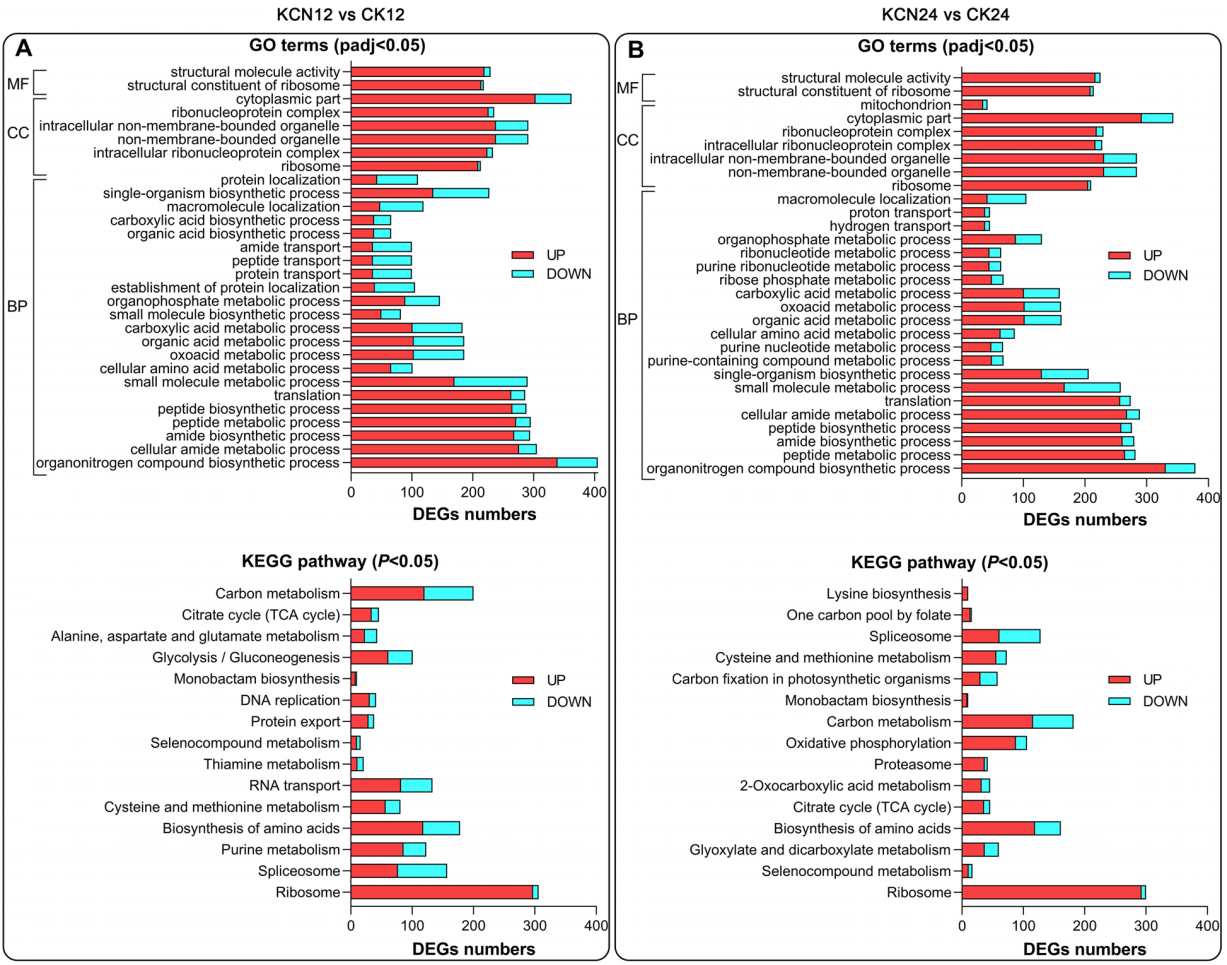
Comparison of GO and KEGG terms with or without cyanide pretreatment. **A** GO terms and KEGG pathway enriched in KCN12 vs. CK12. **B** GO terms and KEGG pathway enriched in KCN24 vs. CK24. The order of GO and KEGG terms are sorted according to the *P* values from small to large, and then the top 30 GO terms and 15 KEGG terms are selected for shown. MF, Molecular Function. CC, Cellular Components. BP, Biological Process

Interestingly, GO and KEGG analysis showed that the DEGs in KCN12 vs. CK12 were significantly enriched in ribosome, spliceosome, biosynthesis of amino acid, and cysteine and methionine metabolism (Fig. 4A). In addition to these pathways, some DEGs in KCN24 vs. CK24 were significantly enriched in the TCA cycle, carbon metabolism, and oxidative phosphorylation pathways (Fig. 4B). Importantly, all the DEGs assigned to ribosome (sly03010) were significantly up-regulated by cyanide pretreatment (Supplementary Fig. S3). Likewise, a majority of DEGs enriched in biosynthesis of amino acid (sly01230) and cysteine and methionine metabolism (sly00270) were significantly up-regulated by cyanide pretreatment (Supplementary Fig. S4). These findings suggest that cyanide pretreatment may help accelerate intracellular biochemical and molecular metabolism.

### Cyanide pretreatment promotes the decomposition of SSP and LEA proteins

Seed storage proteins (SSPs) are proteins that accumulate significantly in developing seeds, whose main function is to act as a storage reserve for nitrogen, carbon, and sulfur. These proteins are rapidly mobilized during seed germination and serve as the major source of reduced nitrogen for growing seedlings [28]. Late embryogenesis abundant (LEA) proteins are defined by the pronounced increase in their expression in embryos during late embryogenesis and their disappearance during subsequent germination stages [29]. Interestingly, it is worth noting that the expression of SSP- and LEA-related genes was significantly down-regulated by cyanide pretreatment (Table 1). The gene expression of SSPs in cyanide-pretreated seeds, including 2S, 11S, and 12S proteins, was 3- to 8-fold lower than that of the control (KCN12 vs. CK12) (Table 1). The differences were more pronounced in KCN24 vs. CK24 (Table 1), indicating that cyanide pretreatment helps mobilize the utilization of stored protein. In addition, the results showed that cyanide pretreatment also significantly down-regulated the gene expression of LEAs (Table 1). Compared with the control seeds, the expression of most LEA genes in cyanide-pretreated seeds was decreased by 4 to 16 times (Table 1), indicating that LEA proteins were involved in cyanide-mediated tomato seed germination.

**Table 1:** DEGs related to storage protein and late embryogenesis abundant protein

Gene id	KCN12 vs. CK12	KCN24 vs. CK24	Gene description	Note
	Log_2_FC			
101,252,924	−3.01	−5.07	11S globulin seed storage protein 2-like	SSP
101,258,989	− 2.39	−5.07	12S seed storage protein CRA1-like	SSP
101,253,730	−1.53	−2.94	12S seed storage protein CRD	SSP
101,256,608	−1.67	−4.99	2S sulfur-rich seed storage protein 1-like	SSP
101,268,495	−3.15	−5.18	2S sulfur-rich seed storage protein 2	SSP
101,268,783	−1.58	−3.17	2S sulfur-rich seed storage protein 2-like	SSP
101,256,744	−4.39	−4.60	vicilin-like seed storage protein At2g18540	SSP
101,246,214	−2.59	−3.56	vicilin-like seed storage protein At2g28490	SSP
544,157	−0.42	−0.39	late embryogenesis (Lea)-like protein	LEA
100,750,252	−1.93	−1.30	late embryogenesis abundant protein	LEA
101,263,544	−0.92	−3.29	late embryogenesis abundant protein 31-like	LEA
101,249,859	−3.02	−2.54	late embryogenesis abundant protein 31-like	LEA
101,250,176	−2.76	−2.51	late embryogenesis abundant protein 46-like	LEA
101,249,875	−3.01	−2.10	late embryogenesis abundant protein 46-like	LEA
101,243,796	−1.84	−2.05	late embryogenesis abundant protein 6	LEA
104,645,830	−1.76	−1.64	late embryogenesis abundant protein At3g53040-like	LEA
101,250,584	−4.12	−4.01	late embryogenesis abundant protein D-29	LEA
101,244,993	−2.66	−3.79	late embryogenesis abundant protein D-34-like	LEA
101,265,270	−4.04	−4.12	late embryogenesis abundant protein ECP63	LEA
104,649,436	−1.69	−1.67	late embryogenesis abundant protein M17-like	LEA

The adjusted *P* values of all data are less than 0.05

*SSP* Seed storage protein, *LEA* Late embryogenesis abundant protein, *Log*_*2*_*FC* Log2foldchange

### Cyanide pretreatment accelerates glucose metabolism and energy conversion

**Fig. 5:**
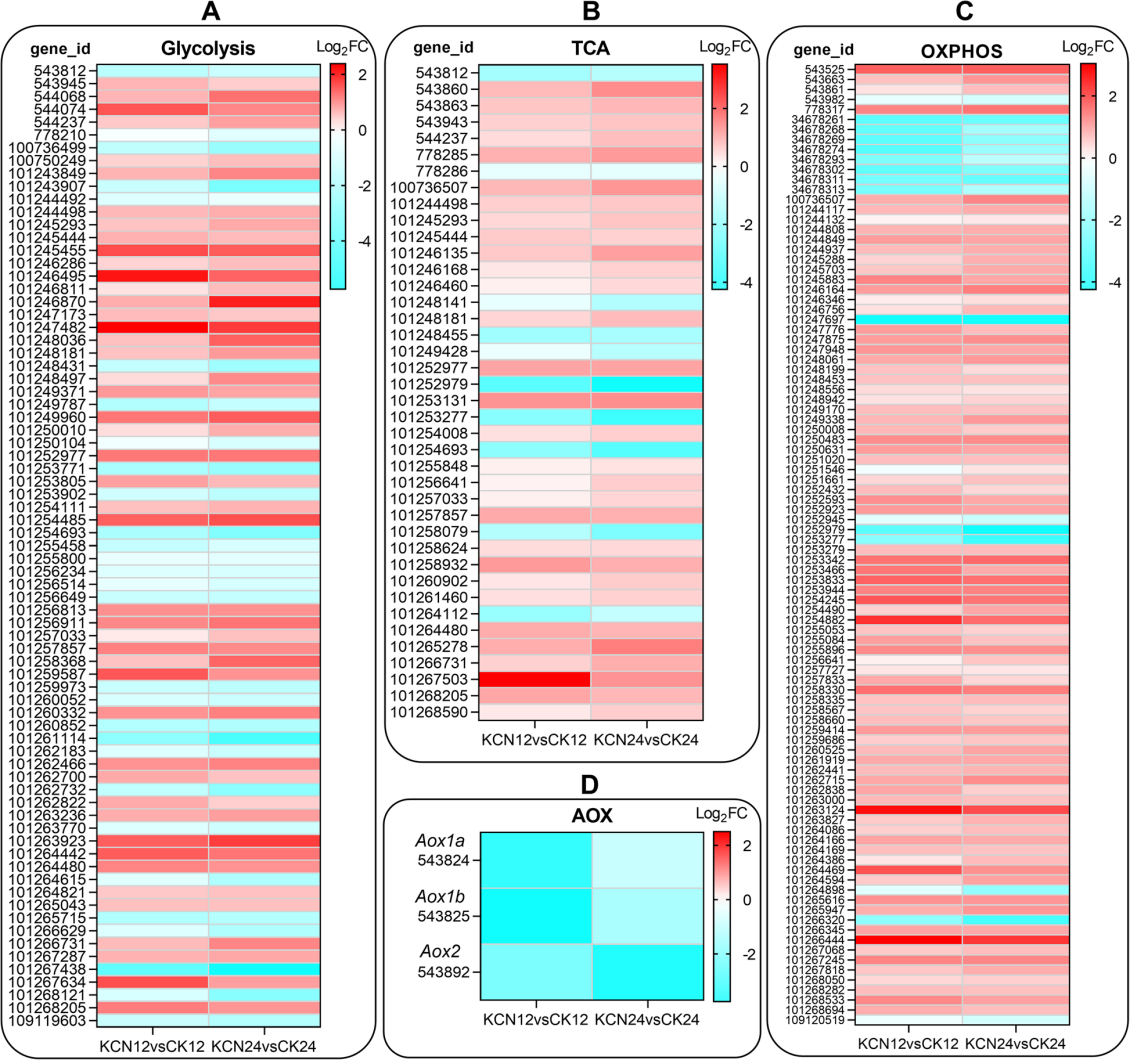
Expression profile of glucose metabolism and cell respiration related genes in response to cyanide pretreatment. Heatmap shows the expression of genes related to glycolysis (**A**), TCA (**B**), oxidative phosphorylation (**C**) and AOX (**D**) pathways. FC, fold change. DEGs, differentially expressed genes. TCA, tricarboxylic acid cycle. AOX, alternative oxidase

Since GO and KEGG analyses showed that energy metabolism pathways were regulated by cyanide, the DEGs were further analyzed in KCN12 vs. CK12 and KCN24 vs. CK24. As shown in Fig. 5, the DEGs assigned to glycolysis and TCA were significantly up-regulated by cyanide pretreatment, and more pronounced in KCN24 vs. CK24 (Fig. 5A, B). In particular, 96 DEGs assigned to the oxidative phosphorylation pathway were enriched in both KCN12 vs. CK12 and KCN24 vs. CK24, and most of them showed up-regulated gene expression after cyanide pretreatment (Fig. 5C). However, it should be noted that the cyanide-resistant pathway, i.e., AOX pathway related gene expression was down-regulated after cyanide pretreatment (Fig. 5D). These findings indicate that pretreatment with a lower concentration of cyanide (10 μM KCN) did not damage cellular respiration but helped to accelerate glucose metabolism and energy conversion.

### Cyanide pretreatment inhibits ABA but promotes GA biosynthesis and perception

**Fig. 6:**
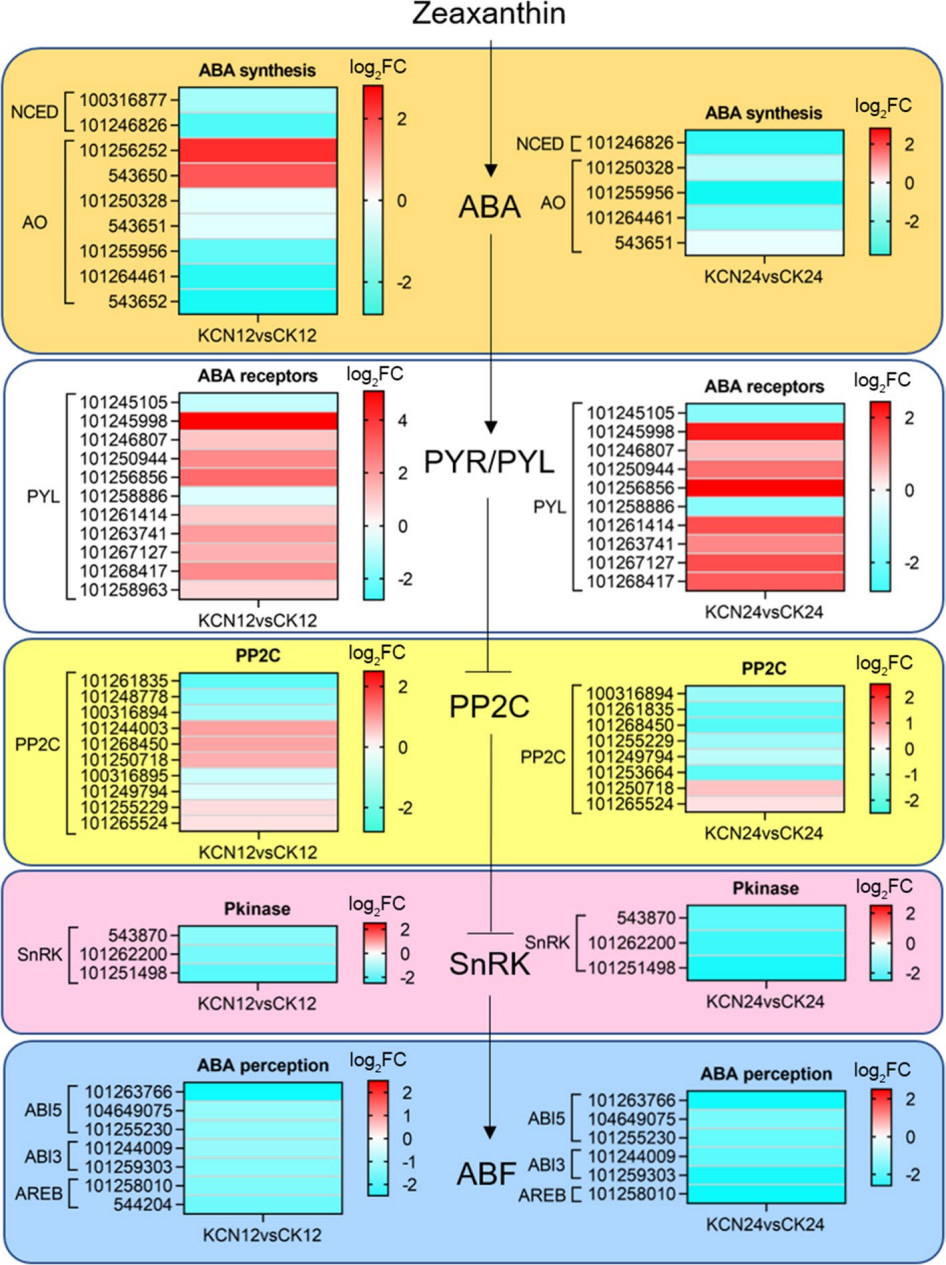
Expression profile of ABA biosynthesis and signal transduction related genes in response to cyanide pretreatment. FC, fold change. DEGs, differentially expressed genes. AO, ABA aldehyde oxidase. NCED, 9-cis-epoxycarotenoid dioxygenase. PYL, pyrabactin resistance 1-like. PP2C, protein phosphatase 2C. SnRK, SNF1-related protein kinase

**Fig. 7:**
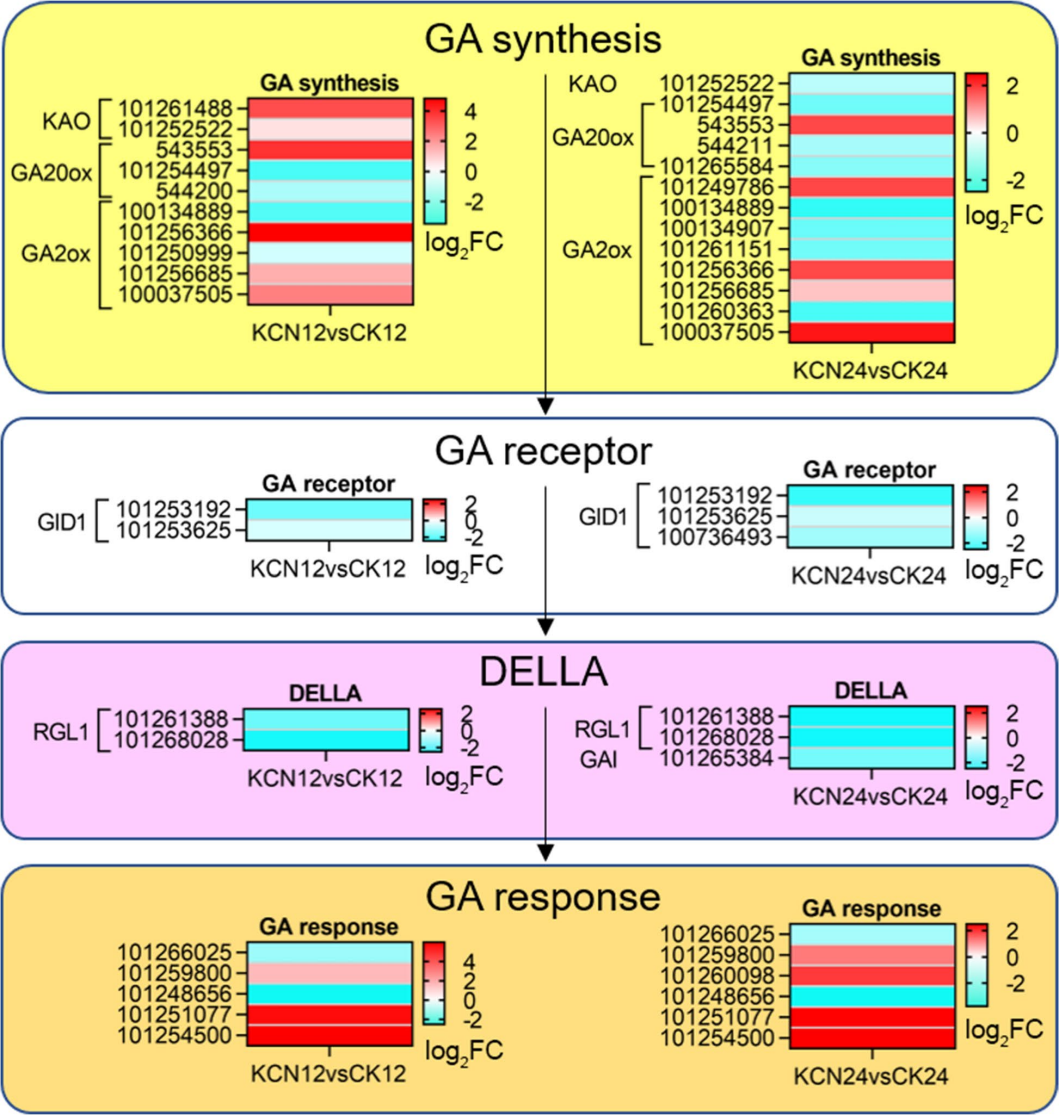
Expression profile of GA biosynthesis and signal transduction related genes in response to cyanide pretreatment. FC, fold change. DEGs, differentially expressed genes. KAO, ent-kaurenoic acid oxidase. GA, gibberellin. GA20ox, gibberellin 20 oxidase. GA2ox, gibberellin 2 oxidase. GID1, gibberellin insensitive DWARF1. RGL1, RGA-like1. GAI, GA-insensitive

Considering that plant hormones are involved in the regulation of seed germination, the DEGs in KCN12 vs. CK12 and KCN24 vs. CK24 were further analyzed to reveal the effects of cyanide pretreatment on hormone biosynthesis and perception. The results showed that cyanide pretreatment down-regulated the gene expression of ABA biosynthesis and signal transduction (Fig. 6), but up-regulated the gene expression of GA biosynthesis and signal transduction (Fig. 7). As shown in Fig. 6, the gene expression of key enzymes of ABA biosynthesis such as 9-cis-epoxycarotenoid dioxygenase (NCED) and abscisic-aldehyde oxidase (AO) was down-regulated after cyanide pretreatment, especially in KCN24 vs. CK24. In addition, the gene expression of protein kinases such as SNF1-related protein kinase (SnRK) and ABA responsive factors including ABA-insensitive 3 (ABI3) and ABA-insensitive 5 (ABI5) was also significantly down-regulated by cyanide pretreatment in both KCN12 vs. CK12 and KCN24 vs. CK24 (Fig. 6). Moreover, it is worth noting that cyanide pretreatment down-regulated the gene expression of DELAY OF GERMINATION1 (DOG1) (Table 2), which is the main regulator of dormancy and was recently identified as a unique ABA signal component in seeds [10, 11, 30].

**Table 2:** Gene expression related to DELAY OF GERMINATION 1 (DOG1)

Gene id	KCN12 vs. CK12	KCN24 vs. CK24	Gene description
	Log_2_FC		
104,646,037	−0.384	−1.185	protein DELAY OF GERMINATION 1-like
101,244,373	1.939	0.885	protein DELAY OF GERMINATION 1-like

*Log*_*2*_*FC* Log2foldchange

The adjusted *P* values of all data are less than 0.05

Conversely, the expression of genes related to GA biosynthesis such as ent-kaurenoic acid oxidase (KAO), was significantly up-regulated by cyanide pretreatment, especially after cyanide pretreatment for 12 h (KCN12 vs. CK12) (Fig. 7). Moreover, the expression of the gibberellin 20 oxidase-1 (GA20ox-1, gene ID: 543553) gene was significantly up-regulated (log_2_FC = 3.84), but the gene expression of gibberellin 2 oxidase (GA2ox, gene ID: 100134889) was obviously down-regulated (log_2_FC = − 2.45) by cyanide pretreatment for 12 h, and this regulatory difference was maintained for the next 12 h (KCN24 vs. CK24) (Fig. 7), further indicating that cyanide pretreatment was involved in enhancing GA biosynthesis. Notably, the results showed that the expression of DELLAs, negative regulators of GA signaling, was down-regulated by cyanide (Fig. 7). In comparison, cyanide pretreatment promoted the expression of GA response related genes (Fig. 7). Taken together, these findings suggest that cyanide pretreatment mediates the inhibition of ABA biosynthesis and perception but promotes the effects of GA.

### Effects of cyanide pretreatment on the biosynthesis and perception of other hormones

**Fig. 8:**
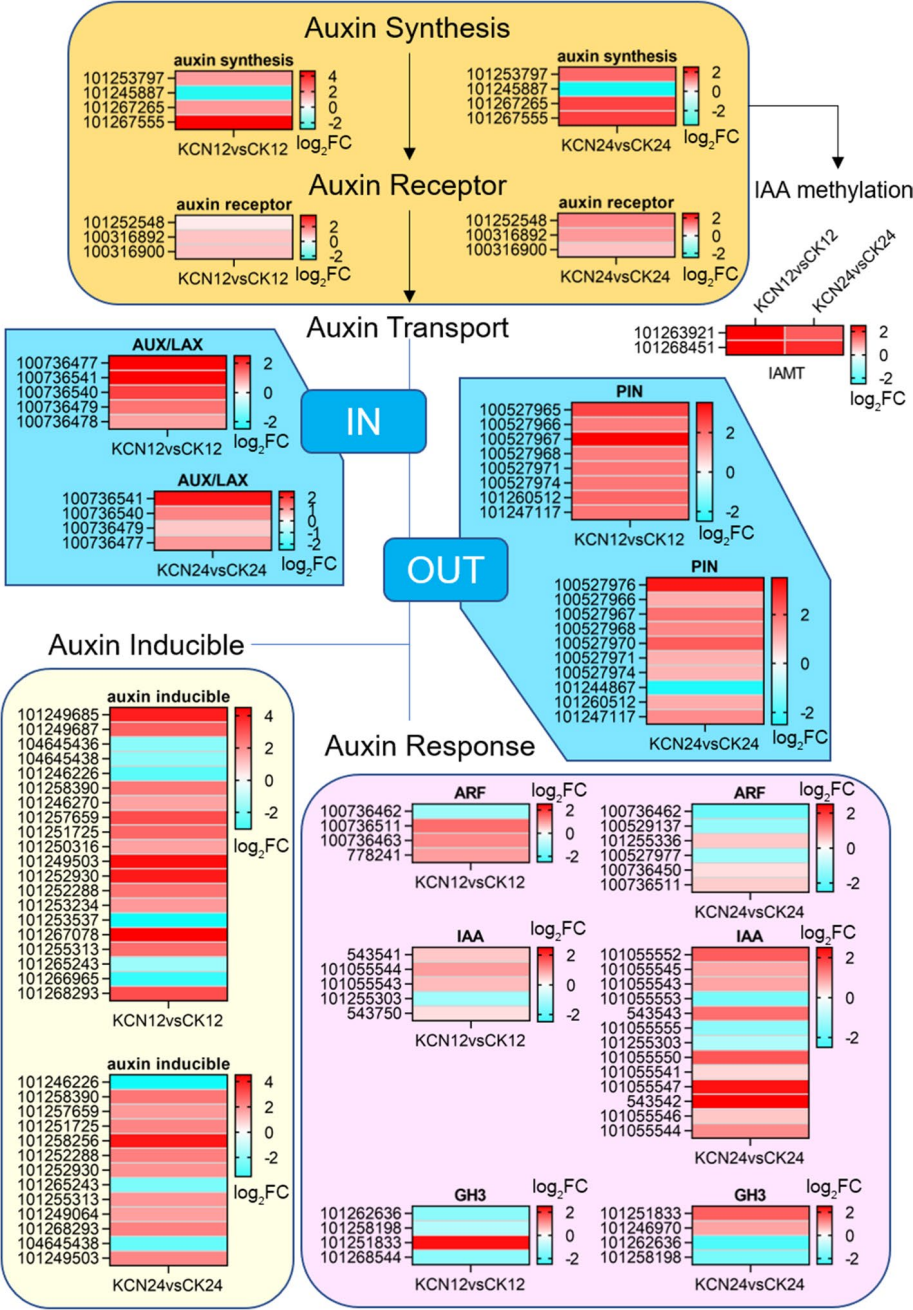
Expression profile of auxin biosynthesis and signal transduction related genes in response to cyanide pretreatment. FC, fold change. DEGs, differentially expressed genes. IAA, indole-3-acetic acid. GH3, GRETCHEN HAGEN 3. ARF, auxin response factor

In addition to ABA and GA, transcriptome analysis showed that cyanide pretreatment was involved in regulating the biosynthesis and perception of auxin, cytokinin (CTK), ethylene (ETH), salicylic acid (SA), and jasmonic acid (JA). As shown in Fig. 8, the expression of auxin biosynthesis, auxin receptor, and auxin transport related genes was up-regulated by cyanide pretreatment. Additionally, a majority of auxin inducible protein and auxin response related genes were up-regulated after cyanide pretreatment (Fig. 8). However, the expression of auxin response factors was up-regulated more obviously after 12 h of cyanide pretreatment (KCN12 vs. CK12), compared with KCN24 vs. CK24 (Fig. 8). Moreover, it should be noted that the gene expression of indole-3-acetic acid-amido synthetase (also known as auxin conjugates) was mainly down-regulated in KCN12 vs. CK12 and mainly up-regulated in KCN24 vs. CK24 (Fig. 8). In addition, the gene expression of indole-3-acetate O-methyltransferase (IAMT) was significantly up-regulated in KCN12 vs. CK12 and KCN24 vs. CK24 (Fig. 8).

After cyanide pretreatment, CTK biosynthesis and perception related genes were mainly up-regulated (Supplementary Fig. S5A). Notably, cyanide pretreatment significantly upregulated the expression of genes related to ETH biosynthesis, but conversely regulated the gene expression of the ETH signal transduction pathway (Supplementary Fig. S5B). For SA and JA, cyanide pretreatment had no significant effects on their biosynthesis and signal transduction, and most genes were down-regulated (Supplementary Fig. S5C, D).

### Hormone determination confirms the regulatory effect of cyanide pretreatment

**Fig. 9:**
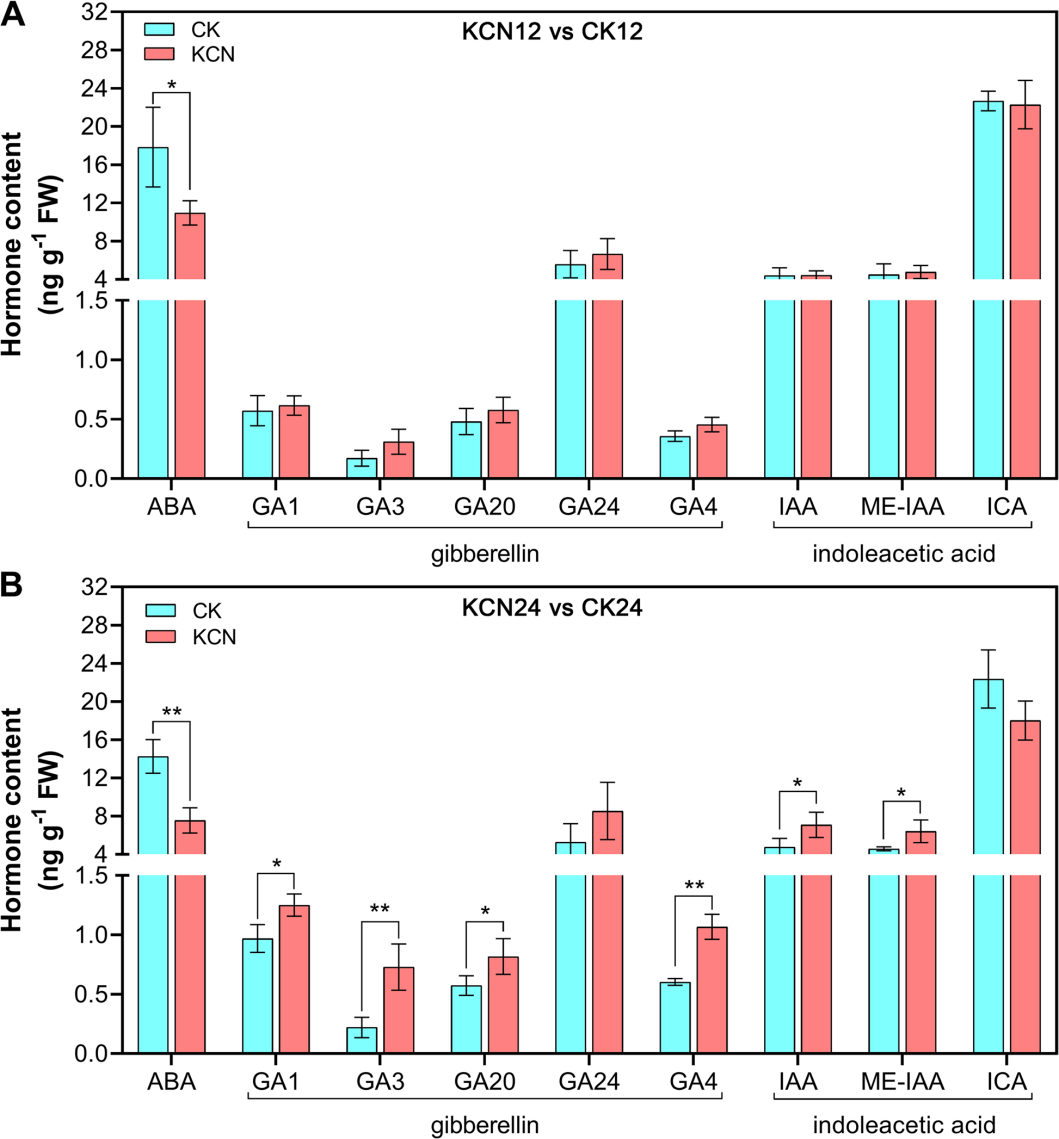
Comparison of ABA, GA, and IAA contents between samples with or without cyanide pretreatment. **A** Quantitative analysis of ABA, GA, and IAA contents after pretreatment with 10 μM KCN for 12 h (KCN12 vs. CK12). **B** After pretreatment with 10 μM KCN for 12 h, the contents of ABA, GA and IAA were quantitatively analyzed for the next 12 h (KCN24 vs. CK24). ME-IAA, methylindole-3-acetic acid. ICA, indole-3-carboxylic acid. Data are the means ±SD of three replicates from three independent experiments. Asterisks represent the significant differences (one-way ANOVA, *P < 0.05 and **P < 0.01) between cyanide-pretreated samples (KCN) and the control (CK)

To further confirm the regulatory effect of cyanide on the biosynthesis of plant hormones, we next measured the hormone content by LC-MS. Since cyanide pretreatment mainly affected ABA, GA, and auxin based on the transcriptome results, we next measured the changes in the content of these hormones. As shown in Fig. 9, the ABA content was markedly inhibited by cyanide pretreatment, which was more pronounced in KCN24 vs. CK24. In contrast, GA content was significantly promoted by cyanide pretreatment, especially GA1, GA3, and GA4 in KCN24 vs. CK24 (Fig. 9B). For the auxin content, there were no significant differences in auxin levels after cyanide pretreatment for 12 h (KCN12 vs. CK12) but the contents of indole-3-acetic acid (IAA) and methyl-IAA (ME-IAA) were up-regulated in KCN24 vs. CK24 (Fig. 9). In addition, the content of indole-3-carboxaldehyde (ICA), which is regarded as a decarboxylative product of IAA, showed no difference between cyanide pretreated samples and the control (Fig. 9). Taken together, these results are consistent with the transcriptome analysis.

### Cyanide pretreatment promotes the antioxidant system during seed germination

**Fig. 10:**
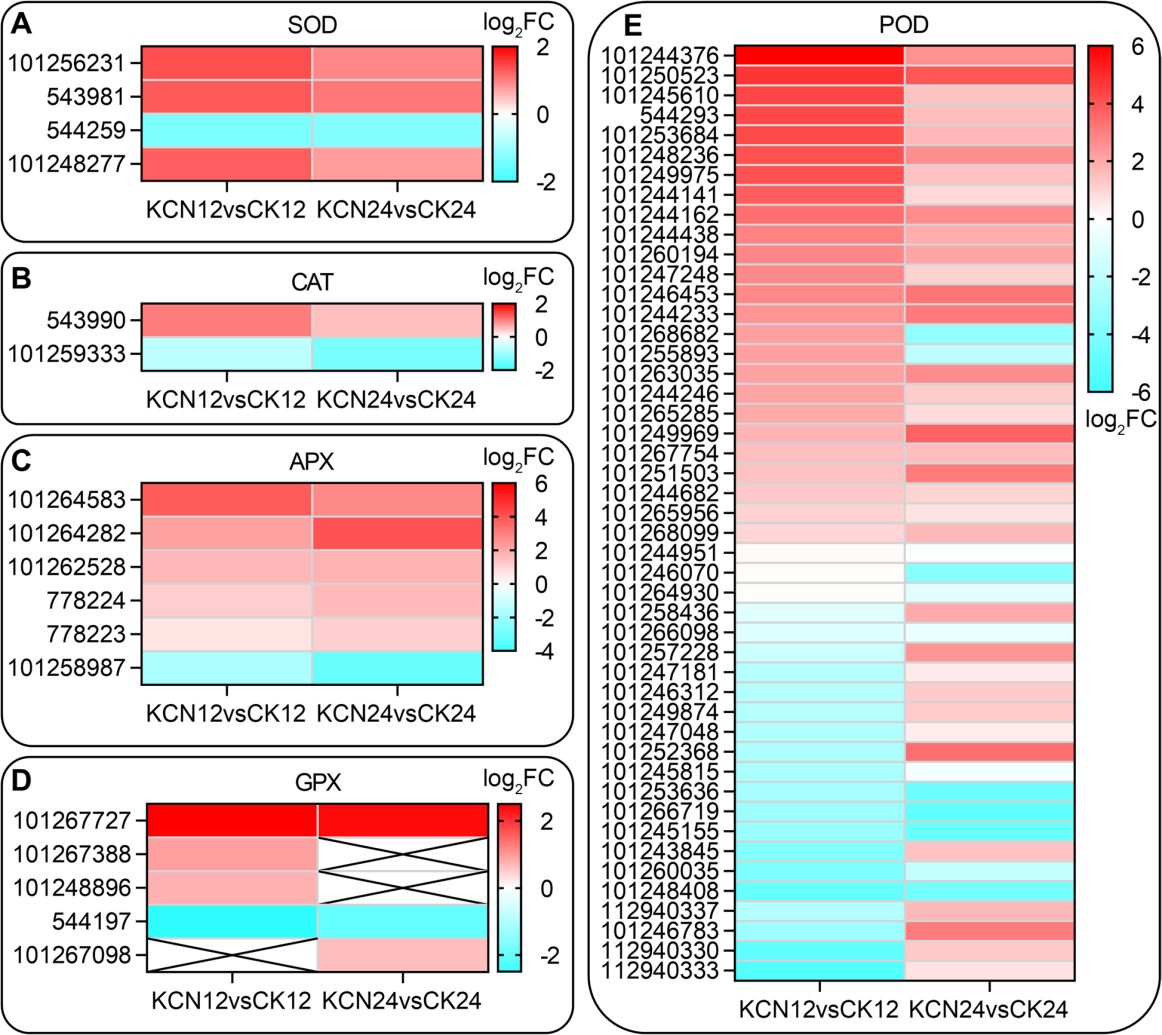
Gene profiles of antioxidants between samples with or without cyanide pretreatment. The gene expression of SOD (**A**), CAT (**B**), APX (**C**), GPX (**D**), and POD (**E**) were analyzed and compared by heatmap diagram. FC, fold change

As mentioned above, there was no excessive accumulation of ROS produced in the cyanide-pretreated seeds compared with the control seeds during germination. This can be explained by the fact that alternative oxidase-mediated cyanide-resistant respiration reduces respiratory depression, or that there was an increase in regulatory genes for anti-oxidant enzymes. As shown in Fig. 10, the antioxidant system, including SOD-, POD-, CAT-, GPX-, and APX- related genes, was regulated by cyanide pretreatment. Notably, the expression of the majority of SOD, POD, APX, and GPX genes was significantly up-regulated after cyanide pretreatment for 12 h (KCN12 vs. CK12), and the following 12 h (KCN24 vs. CK24) (Fig. 10). Therefore, it is apparent that cyanide pretreatment promoted the antioxidant system of tomato seeds, which was beneficial to inhibit the excessive burst of ROS during seed germination.

## Discussion

Seed germination is a crucial process that influences crop yield and quality. Therefore, understanding the molecular aspects of seed dormancy and germination is of great significance for the improvement of crop yield and quality [1]. In this study, we found that a lower concentration of cyanide pretreatment was beneficial for tomato seed germination, especially when cyanide was applied at 10 μM for 12 h (Fig. 1). It is worth noting that cyanide pretreatment promoted seed germination rates and enhanced seed respiration including both total respiration and cyanide-resistant respiration. Transcriptome analysis also showed that cyanide pretreatment affected energy metabolism and cross-talked with the major hormones regulating tomato seed germination.

It is well known that ROS, such as H_2_O_2_, play a pivotal role in the regulation of seed germination [31]. However, it was proposed that ROS homeostasis is critical for controlling the ability of seeds to germinate [32]. Interestingly, our study showed that cyanide pretreatment accelerated the generation of ROS instead of its accumulation (Fig. 2C), indicating that the involvement of ROS levels in regulating seed germination should be restricted to a critical range [32]. We hypothesized that the early production of ROS was related to the mitochondrial respiration promoted by cyanide, because a large number of studies have shown that ROS are primarily produced in the mitochondria, especially during seed germination [33]. Some previous studies demonstrated that cyanide-mediated seed germination was associated with a marked increase in ROS content during germination of apple seeds and sunflower seeds [16, 24]. To explain this difference, it may be attributed to the fact that a lower concentration of cyanide (10 μM) was applied in this study, whereas a significantly higher concentration of cyanide (such as 1 mM) was applied to the seeds in previous studies [16, 26, 27]. Second, cyanide pretreatment induced the expression of antioxidant enzyme-related genes (Fig. 9). Besides, this may be caused by differences in the germination characteristics of different seeds. It has often been reported that ROS have dual roles in plant development including seed germination. Excessive ROS are detrimental to seed germination and if mitochondrial ROS play a role in seed germination, their production must be controlled and regulated [31]. Therefore, this can also explain why the low-concentration cyanide pretreatment for a shorter time is more conducive to seed germination in the present study.

In addition to ROS, there are many factors involved in controlling seed germination, including plant hormones. The hormonal signaling of ABA and GA has been demonstrated to control seed dormancy and seed germination, respectively. In this study, we found that cyanide pretreatment markedly down-regulated the expression of ABA biosynthesis- and perception-related genes. In contrast, the expression of GA biosynthesis- and perception-related genes was up-regulated after cyanide pretreatment. With respect to ABA, genes including *NCED*, *AO*, *SnRK* and *ABF* were significantly down-regulated after cyanide pretreatment. It has been indicated that ABI3 and ABI5 are transcription factors that mediate ABA responses in seed dormancy from a range of species and they are negative regulators in regulating seed germination [4, 34]. Here, we found that cyanide pretreatment significantly down-regulated the gene expression of *ABI3* and *ABI5*, which may be one of the reasons for cyanide-stimulated tomato seed germination. In addition, it is possible that cyanide controls the ABA response by inhibiting the expression of SnRK family protein kinases, which are the key positive regulators of ABA signaling [35]. Similarly, what caught our attention was that cyanide pretreatment inhibited the expression of the *DOG1* gene (Table 2), which may be one of the main reasons for the obstruction of ABA signaling. As stated in previous studies, DOG1 is a master regulator of ABA-mediated seed dormancy, which can block the action of PP2C and enhance ABA signaling [10, 30]. In contrast, GA biosynthesis and response were enhanced by cyanide pretreatment. Importantly, the expression of negative regulators of the GA signaling pathway, DELLAs including RGL1 and GAI, was significantly down-regulated by cyanide pretreatment. In fact, the detection and comparison of ABA and GA contents also confirmed the positive or negative effects of cyanide on the two, respectively (Fig. 9). Together, it appears that cyanide stimulated tomato seed germination by reducing ABA biosynthesis and signal transduction while increasing GA levels and perception.

Among the possible mechanisms of cyanide action in seed germination, its interplay with ethylene metabolism has often been cited, mainly because cyanide is a co-product of ethylene biosynthesis [17, 27]. Ethylene is implicated in the promotion of germination of non-dormant seeds of many species, which promotes seed germination by counteracting ABA effects [7, 36]. In addition, it has been proposed that cyanide-promoted seed germination is associated with ethylene stimulation (feedback effect) and signal transduction [26, 37]. The findings from Oracz [27] demonstrated that the release of sunflower seed dormancy by cyanide was coupled with the ethylene response factor, ERF1. Interestingly, our study showed that, during tomato germination, cyanide pretreatment significantly enhanced the expression of ethylene biosynthesis-related genes such as *ACS* and *ACO* but weakened the gene expression of ethylene signal transduction-related genes, including *ERF1* (Supplementary Fig. S5). We speculate that the regulation of cyanide may be different during the germination of different seeds. However, previous studies [26, 27, 37] and our studies have shown that ethylene is involved in cyanide-mediated seed germination. Furthermore, cyanide-induced ethylene biosynthesis may help mobilize energy metabolism and cross-talk with other hormones. As the review of Matilla (2000) stated that ethylene action may also be related to stimulating respiration and amino acid accumulation in primed seeds [38].

In this study, the gene expression of seed storage proteins (SSPs) and late embryogenesis abundant (LEA) proteins was significantly down-regulated by cyanide pretreatment, indicating that cyanide action is also involved in regulating energy mobilization. It is generally accepted that SSPs accumulate significantly in developing seeds and are rapidly mobilized during seed germination [39, 40]. Likewise, LEA proteins are enriched in the embryo during late embryonic development and disappear in the subsequent germination stage [29]. Previous studies have shown that SSPs and LEA proteins respond to ABA signaling during seed development. For example, the ABI3 and B3 factors FUS3 (ABI3 homolog-like protein) have been demonstrated to directly activate SSPs gene expression in *Arabidopsis thaliana* [41]. In addition, it has been observed that the gene expression of LEA proteins is regulated by ABI3, which is suppressed in *abi3* mutants [29]. In contrast, GA has been proposed to stimulate the mobilization of SSPs and LEA proteins by suppressing the expression of these genes while promoting the transcription of hydrolysis-related genes [42]. Consequently, these findings led to the hypothesis that cyanide promoted tomato seed germination by accelerating the mobilization of storage reserves, thus promoting the supply of energy and nutrients to help seed germination. However, whether the effect of cyanide on the storage substance is direct (i.e., as a signal molecule) or indirect (i.e., by regulating ABA and GA signals) remains to be confirmed by further studies in the future.

## Conclusions

In summary, our study suggests that cyanide-stimulated tomato seed germination occurs in a dose- and time- dependent manner. It is worth noting that the appropriate concentration of cyanide pretreatment contributes to respiration metabolism and the degradation of storage materials required for seed germination. In addition, cyanide may act as a signaling molecule to control the balance of ABA and GA by regulating their biosynthesis and signal perception. Combined with previous studies, we believe that the effective concentration and duration of cyanide differ in controlling the germination of different plant seeds. In any case, our research provides useful resources for further understanding and elucidating the molecular mechanism of cyanide-mediated seed germination.

## Methods

### Plant materials and chemical treatment

In this study, the seeds of tomato (*Solanum lycopersicum* cv. Alisa Craig) were used, which originally obtained from Thompson and Morgan (https://www.thompson-morgan.com/).

For germination, seeds were surface-sterilized with 0.1% mercury bichloride for 10 min and then were washed with ddH_2_O for at least three times. After that, some seeds were pretreated with different concentrations of potassium cyanide (5, 10, 50, 100 μM) for 6 h, 12 h, and 24 h at 25 °C. The other seeds were pretreated with ddH_2_O (defined as controls) under the same conditions. In each pretreatment experiment, approximately 100 sterilized seeds were used. All pretreatments had three biological replicates in each experiment, and three or more independent experiments were carried out for the whole study.

After pretreatment, cyanide was removed and the seeds were placed on sterile filter paper moistened with deionized water for germination and growth under the conditions of 16 h of light (approx. 150 μmol m^− 2^ s^− 1^) and 8 h of dark, at 25 °C, 70% relative humidity.

### Germination test

Germination was scored over time, and the initiation of the germination process was calculated from the moment that the seeds were treated with cyanide or water; a seed was considered germinated when the radicle protruded from the teguments by 1 ~ 2 mm. The germination percentage (%) and germination speed (T_50_) were computed as described previously [43]. T_50_, time to obtain 50% of germinated seeds.

### Respiration measurement

Respiration of seeds was performed as described previously [44] with some modification. In this experiment, ten seeds were collected and transferred into air-tight cuvettes containing 2 ml of phosphate buffered saline (pH 7.5), and oxygen uptake was measured as a decrease of oxygen concentration in the dark using a Clark-type electrode (Chorolab-2; Hansatech, King’s Lynn, UK). Total respiration rate (*V*_t_) was achieved when the measurement without any inhibitors. Then, 2 mM salicylhydroxamic acid (SHAM, an AOX inhibitor) [45, 46], was added to inhibit cyanide-resistant respiration rate (*V*_alt_), and the values of cytochrome pathway respiration (*V*_cyt_) and residual respiration rate (*V*_res_) were obtained. *V*_alt_ = *V*_t_-*V*_cty_-*V*_res_.

### H_2_O_2_ content estimation

The H_2_O_2_ content was measured as described previously [47]. In this experiment, ten seeds were homogenized with liquid nitrogen and then mixed with 5 mL 0.1% (w/v) trichloroacetic acid (TCA). The homogenate was centrifuged at 12000 g for 20 min at 4 °C. In addition, 0.5 mL of the supernatant was added to 0.5 mL 10 mM potassium phosphate buffer (pH 7.0) and 1 mL 1 M KI. The absorbance of the supernatant was read at 390 nm.

### HPLC for plant hormone determination

The plant hormones include GA, ABA, and IAA were measured by HPLC. For GA extraction and measurement, samples were ground carefully and 0.2 g of the powdered sample was extracted overnight at 4 °C with 1.5 mL 70% (V/V) acetonitrile. After vortex for 30 s and centrifugation at 14,000 rpm for 10 min, the supernatants (1.0 mL) were collected and then evaporated to dryness under nitrogen gas stream at room temperature, constituted in 100 μL 80% (V/V) methanol, diluted to 800 μl with water. The extracts were passed through the SPE cartridge (CNWBOND Carbon-GCB SPE Cartridge, 200 mg, 3 mL; Anpel, Shanghai, China) and evaporated to dryness under nitrogen gas stream at room temperature. Following the sample were reconstituted in 200 μl 80% (V/V) methanol and filtrated (PTFE, 0.22 μm; Anpel, Shanghai, China) before LC–MS/MS analysis [48].

For ABA and IAA extraction and measurement, 50 mg samples were ground with liquid nitrogen and extracted with 0.5 mL methanol/water/formic acid (15:4:4, V/V/V) at 4 °C. The extract was vortexed for 10 min and centrifuged at 14,000 rpm for 5 min at 4 °C. The supernatants were collected and repeated the steps above. The combined extracts were evaporated to dryness under nitrogen gas stream, reconstituted in 80% methanol (V/V), ultraphoniced (1 min) and filtrated (PTFE, 0.22 μm; Anpel, Shanghai, China) before LC-MS/MS analysis.

All of the standards were purchased from Olchemim Ltd. (Olomouc, Czech Republic) and Sigma (St. Louis, MO, USA). More details about the LC-MS/MS analysis can be found in Supplementary Methods.

### RNA extraction and transcriptome sequencing

For RNA-SEQ analysis, tomato seeds pretreated with 10 μM KCN for 12 h (labeled KCN-12) were used in this study. In addition, after cyanide pretreatment, the seeds were placed under normal conditions for another 12 h (labeled KCN-24) were also used for analysis. The seeds without cyanide pretreatment for 12 h (CK-12) and 24 h (CK-24) were used as the control. Subsequent handling of tomato samples includes extraction, purification, analysis and sequencing of total RNA performed by Novogene Bioinformatics Technology Co. Ltd. (Beijing, China). Sequencing libraries were generated using NEBNext® Ultra™ RNA Library Prep Kit for Illumina® (NEB, USA) according the manufacturer’s instructions, and index codes were added to attribute sequences to each sample.

### Quantification of gene expression levels

HISAT2 was used to count the number of reads mapped to each gene. In addition, the FPKM (fragments per kilobase of exon model per million mapped reads) of each gene was calculated based on the length of the gene and the number of reads mapped to the gene.

### Differential expression analysis

Differential expression analysis was performed using the DESeq2 R package (1.16.1). DESeq2 provides statistical routines for determining differential expression in digital gene expression data using a model based on the negative binomial distribution. The resulting *P*-values were adjusted using the Benjamini and Hochberg’s approach for controlling the false discovery rate. Genes with an adjusted *P* < 0.05 found by DESeq2 were assigned as differentially expressed. In addition, the gene IDs are shown in the table and figures, and the gene name is the gene IDs that contain the prefix “LOC”.

### GO and KEGG enrichment analysis

Gene Ontology (GO) enrichment analysis of differentially expressed genes was implemented by the cluster Profiler R package, in which gene length bias was corrected. GO terms with corrected *P*-value less than 0.05 were considered significantly enriched by differential expressed genes.

Kyoto Encyclopedia of Genes and Genomes (KEGG) is a database resource for understanding high-level functions and utilities of the biological system, such as the cell, the organism and the ecosystem, from molecular-level information, especially large-scale molecular datasets generated by genome sequencing and other high-through put experimental technologies (http://www.genome.jp/kegg/). We used cluster Profiler R package to test the statistical enrichment of differential expression genes in KEGG pathways.

### Real-time quantitative PCR analysis

In order to validate the results from transcriptome sequencing analysis, part of genes was confirmed by quantitative real-time PCR (qRT-PCR) and *Actin* (Accession number: AB158612) gene was used as internal control. All the Primers are listed in Supplementary Material: Table S1. qRT-PCR reactions were prepared with the SYBR Green Master Mix Reagent (Applied Biosystems, MA, USA), following the manufacturer’s instruction. Reactions were carried out in Applied Real-Time System (ABI7500). All samples were performed in triplicate and relative expression levels were calculated using the delta-delta Ct method of the system.

### Statistical analysis

Statistical analysis of the results from three independent experiments. The averages and standard deviations (SD) of all results were calculated, and one-way analysis of variance (ANOVA) were performed to generate *P* values. The difference was considered to be statistically significant when *P < 0.05, ** *P* < 0.01, and *** *P* < 0.001.

## Supplementary Information

**Additional file 1: Table S1.** Primers used for qRT-PCR. **Figure S1.** Hierarchical clustering analysis of the expression profile of each sample. **Figure S2.** Quantitative analysis of the selected tomato genes by RNA-SEQ and qRT-PCR. **Figure S3.** KEGG expression profile of the DEGs assigned to ribosome. **Figure S4.** DEGs related to amino acids biosynthesis and cysteine and methionine metabolism. **Figure S5.** Heatmaps showing the DEGs related to CTK, ETH, SA, and JA biosynthesis and perception.

**Additional file 2:.** Supplementary Materials and Methods.

## Data Availability

The data presented in the study are deposited in the sequence read archive (SRA) repository, accession numbers (SRR13787016–SRR13787027). The data can be viewed through the reviewer link (https://dataview.ncbi.nlm.nih.gov/object/PRJNA705011?reviewer=fvo840u8kdtdj14iei1nms8cit).

## Abbreviations

ABA: Abscisic acid
ABI3: ABA-insensitive 3
ABI5: ABA-insensitive 5
AO: Abscisic-aldehyde oxidase
AOX: Alternative oxidase
BRs: Brassinosteroids
CTK: Cytokinin
DOG1: DELAY OF GERMINATION-1
ETH: Ethylene
GAs: Gibberellins
GA20ox: Gibberellin 20 oxidase
GO: Gene ontology
H_2_O_2_: Hydrogen peroxide
HCN: Hydrogen cyanide
IAA: Indole-3-acetic acid
ICA: Indole-3-carboxaldehyde
JAs: Jasmonates
KAO: Ent-kaurenoic acid oxidase
KCN: Potassium cyanide
KEGG: Kyoto Encyclopedia of Genes and Genomes
LEA: Late embryogenesis abundant
ME-IAA: Methyl-IAA
NCED: 9-cis-epoxycarotenoid dioxygenase
NO: Nitric oxide
PD: Primary dormancy
ROS: Reactive oxygen species
SSP: Seed storage proteins
SA: Salicylic acid
SHAM: Salicylhydroxamic acid
SnRK: SNF1-related protein kinase
*V*_alt_: AOX pathway respiration
*V*_t_: Total respiration
