# Supplementary material for: New insights into the role of cyanide in the promotion of seed germination in tomato
Source: BMC Plant Biol. 2022 Jan 11;22:28. doi: 10.1186/s12870-021-03405-8 (PMC8751275; doi:10.1186/s12870-021-03405-8)
Supplement: Supplementary file 1 — Additional file 1: Table S1. Primers used for qRT-PCR. Figure S1. Hierarchical clustering analysis of the expression profile of each sample. Figure S2. Quantitative analysis of the selected tomato genes by RNA-SEQ and qRT-PCR. Figure S3. KEGG expression profile of the DEGs assigned to ribosome. Figure S4. DEGs related to amino acids biosynthesis and cysteine and methionine metabolism. Figure S5. Heatmaps showing the DEGs related to CTK, ETH, SA, and JA biosynthesis and perception. [file 12870_2021_3405_MOESM1_ESM.docx]

**Supporting data**

**Table S1 Primers for qRT-PCR**

| Gene ID | Forward Primer (5'→3') | Reverse Primer (5'→3') |
| --- | --- | --- |
| 543553 | GCCTCATTGTGATCCAACA | GTGTCGCCTATATTAACCAC |
| 543892 | TAACGGTTGCATATCGTCT | CACCGACCAGAGAATCTCA |
| 544024 | AAATCAAGATGCGTTTCGAC | GAGTGTTTATCTCTTCCGTCT |
| 100134889 | AAGTAAACTCGAATCCGAAGC | AATGTATTCAACCCAACCGAT |
| 100750252 | CAAAGGATAAGGCTTCCGACA | CGCCTTATCCTTAGTAGCTTG |
| 101055547 | TAACAAAGATCCAATCAAGCC | GCAAACTTTGACAAATGCAG |
| 101251626 | TTGTCCAATCAGTTCCGCCAC | GAGTGAGAAGCAGCGGCGGAA |
| 101251725 | AGGAAGAATTTGGGTTCGATC | TATTCACTCGCACATAACGAA |
| 101252924 | TCCAGAACATTTTCCGTGCTT | CTTCATCAGCCCTAATCATGC |
| 101253730 | TATTACTTCTTCAGCCTCGTG | TGTCACCAGCATTGTACCA |
| 101256856 | TTTCTCAAAAGCTGCCACGTC | ATTGTTCAGCCTGTGATCTCC |
| 101263766 | AGGCAATCTTCCATATACTCGT | TTTCCTACAGGTGCAATCCCA |
| 101260631 | AATAGCATAAGATGGCAGAC | GCTTCAGTTAGGAGGACAGG |


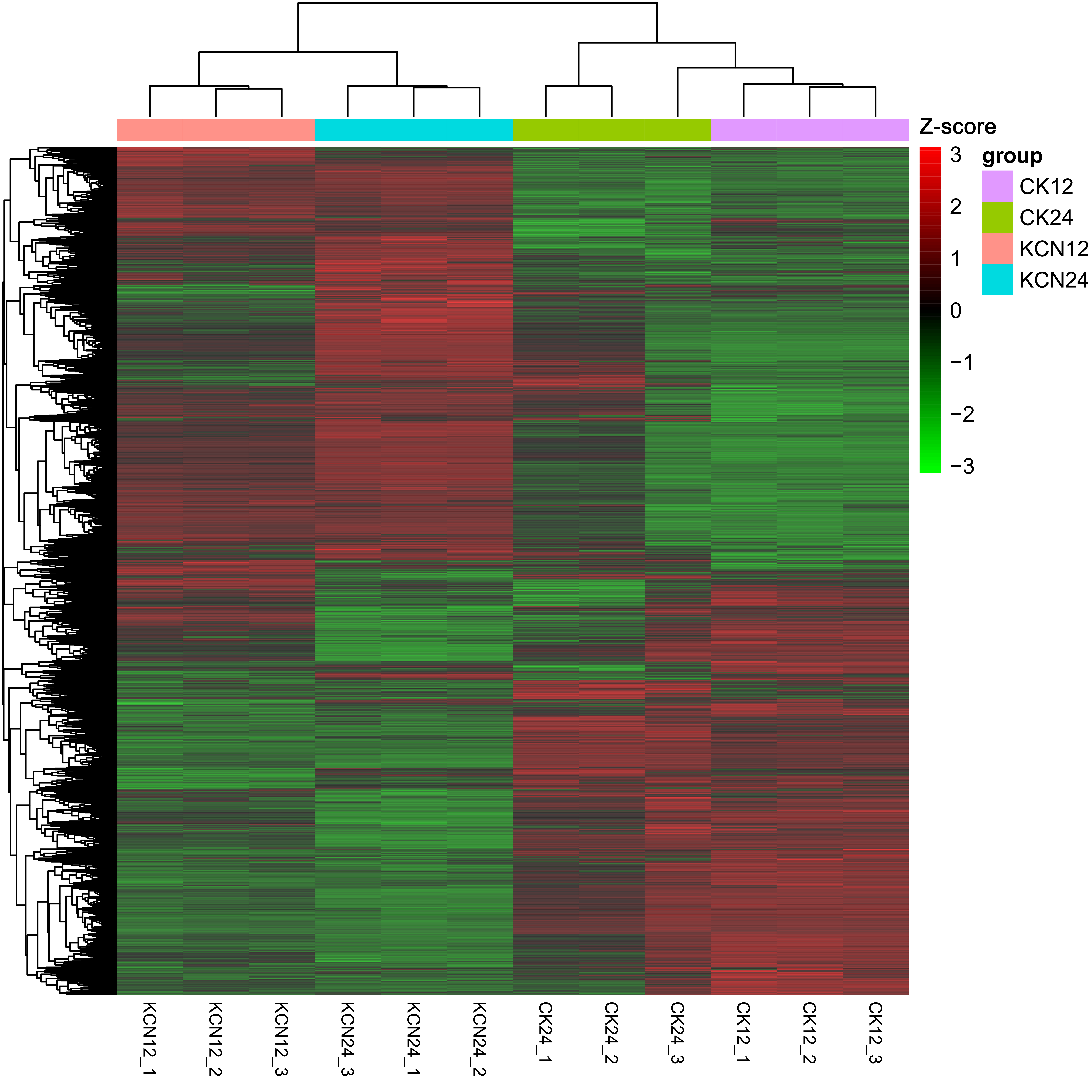


**Fig. S1** **Hierarchical clustering analysis of the expression profile of each sample**. Hierarchical clustering analysis of normalized count data z-scores exhibited by DEGs of each sample at each time point. DEGs, differentially expressed genes.


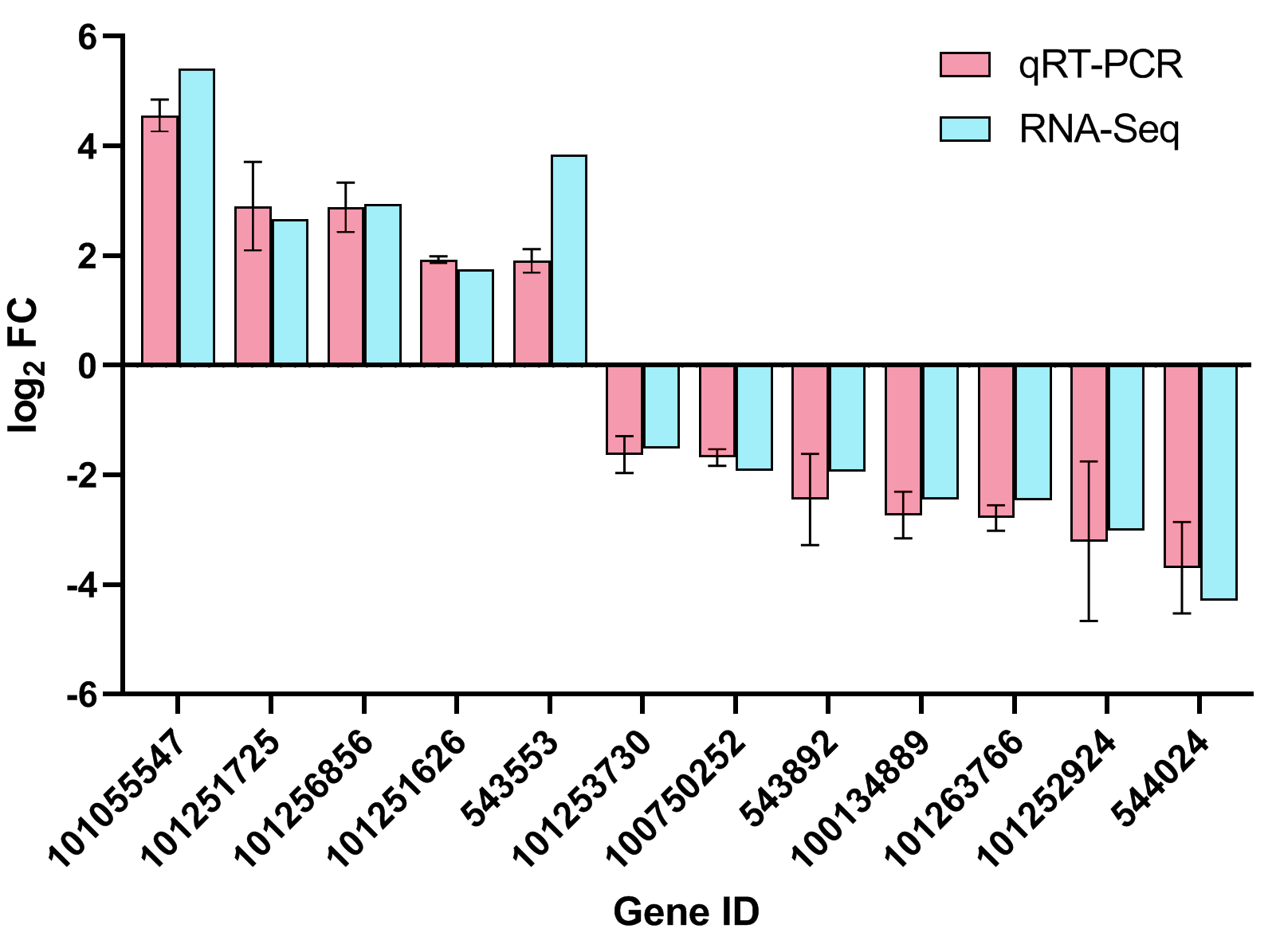


**Fig. S2** **Quantitative analysis of the selected tomato genes by RNA-SEQ and qRT-PCR**. Data are presented as log_2_FC values in comparison with the control. FC, fold change. The RNA-SEQ data are from transcriptome sequencing, while the qRT-PCR data are the mean ± SD of three replicates from three independent experiments.


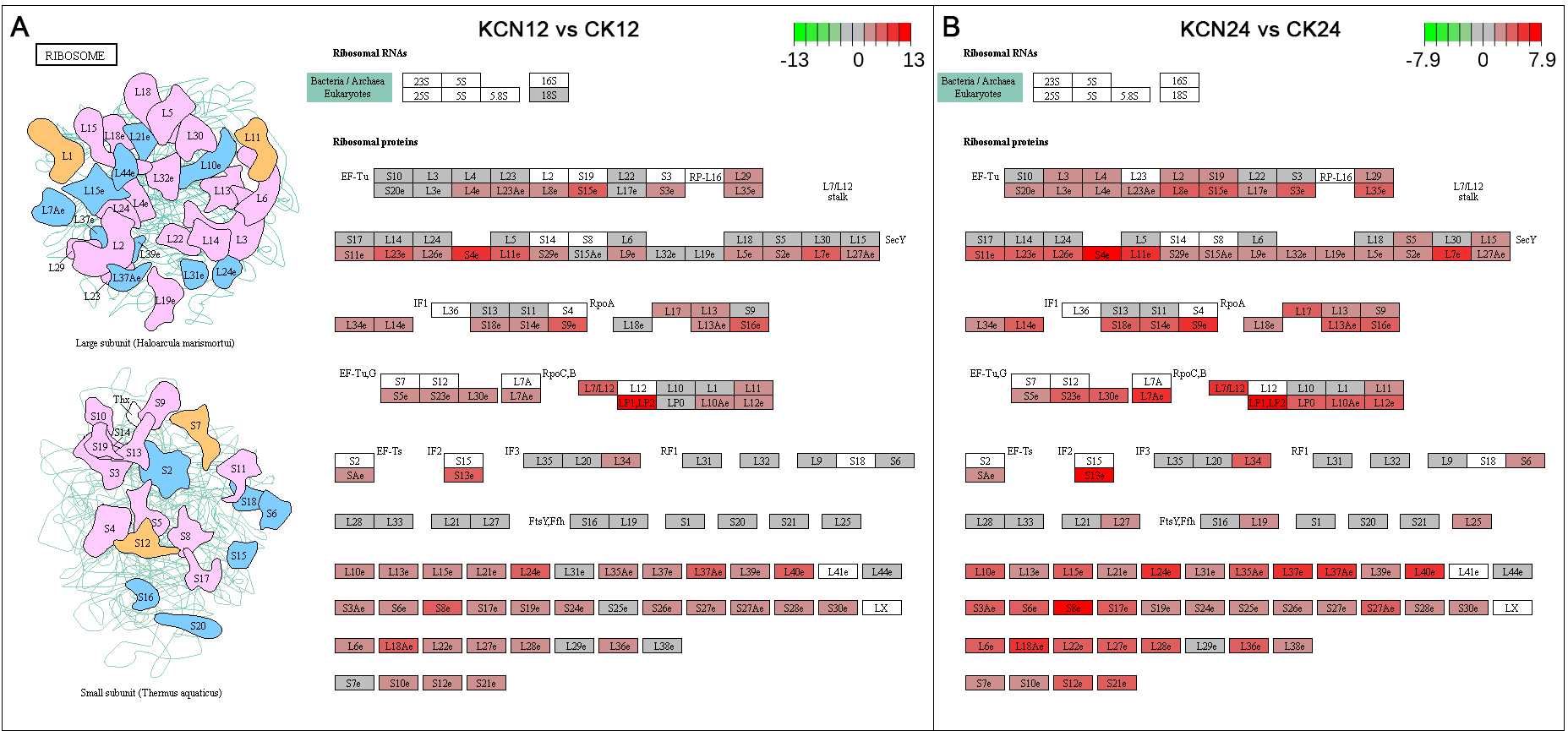


**Fig. S3** **KEGG expression profile of** **the DEGs assigned to ribosome**. (A) Expression profile of DEGs in KCN12 vs. CK12. (B) Expression profile of DEGs in KCN24 vs. CK24. DEGs, differentially expressed genes. EF-Tu, elongation factor thermal unstable Tu, which is a G protein that catalyzes the binding of aminoacyl-tRNA to the A-site of the ribosome. EF-Ts, elongation factor thermal stable Ts, which promotes the transition of EF-Tu from the inactive GDP form to the active GTP form. EF-G, elongation factor, which catalyzes the movement (translocation) of transfer RNA (tRNA) and messenger RNA (mRNA) through the ribosome. IF1, IF2, and IF3, initiation factors. RF1, release factor 1. RpoA,B,C, RNA polymerase A,B,C. L, large subunit. S, small subunit.


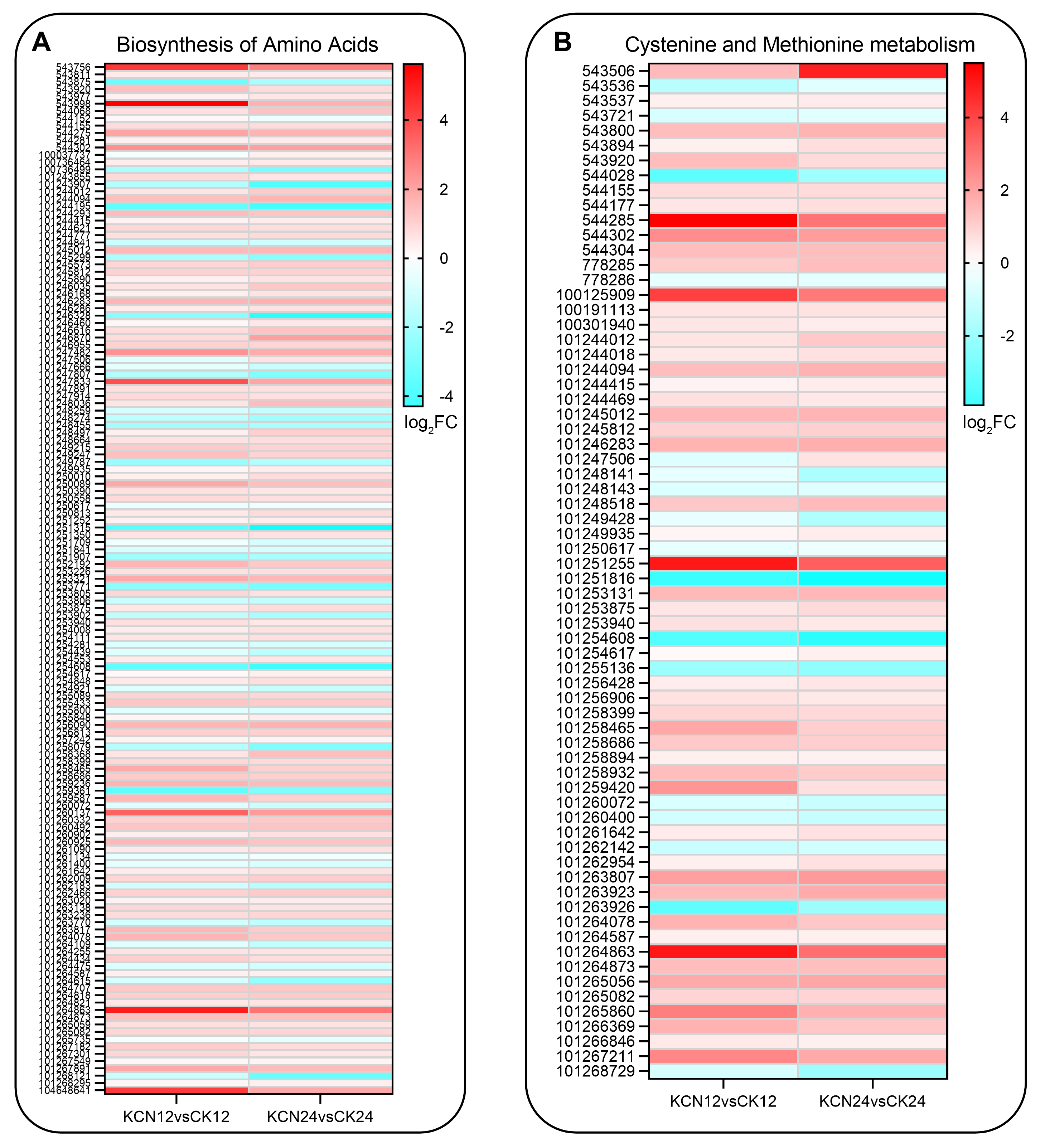


**Figure S4. DEGs related to amino acids biosynthesis and cysteine and methionine metabolism**. (A) Expression profile of amino acids biosynthesis related genes in KCN12 vs. CK12 and KCN24 vs. CK24. (B) Expression profile of cysteine and methionine metabolism related genes in KCN12 vs. CK12 and KCN24 vs. CK24. FC, fold change. DEGs, differentially expressed genes.


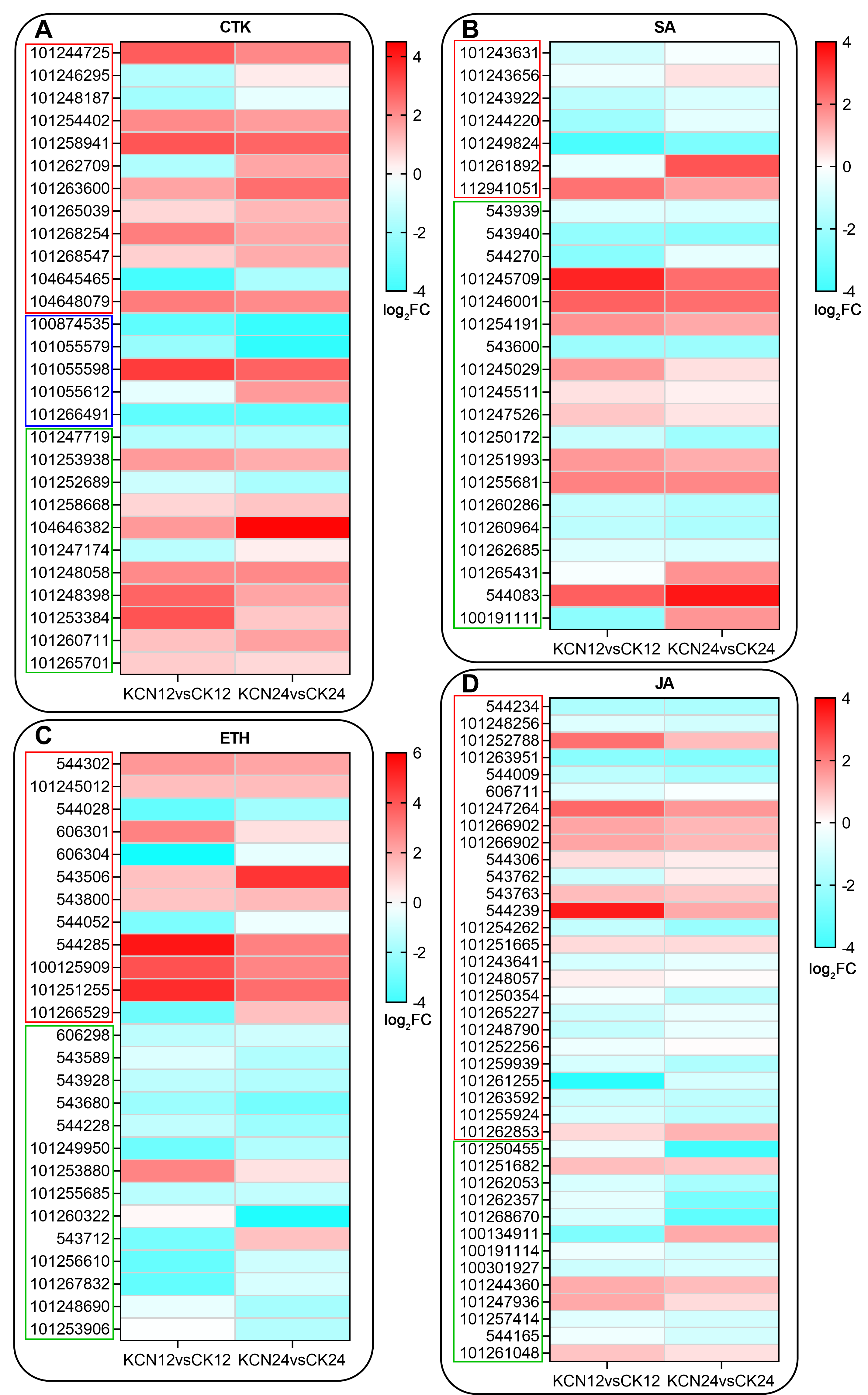


**Figure S5. Heatmaps showing the DEGs related to CTK, ETH, SA, and JA biosynthesis and perception**. (A) Expression profile of the genes related to CTK biosynthesis (red block), degradation (blue block), and perception (green block). (B) Expression profile of the genes related to SA biosynthesis (red block) and perception (green block). (C) Expression profile of the genes related to ETH biosynthesis (red block) and perception (green block). (D) Expression profile of the genes related to JA biosynthesis (red block) and perception (green block). CTK, cytokinin. SA, salicylic acid. ETH, ethylene. JA, jasmonic acid. FC, fold change. DEGs, differentially expressed genes.
