# Supplementary material for: New insights into the role of cyanide in the promotion of seed germination in tomato
Source: BMC Plant Biol. 2022 Jan 11;22:28. doi: 10.1186/s12870-021-03405-8 (PMC8751275; doi:10.1186/s12870-021-03405-8)
Supplement: Supplementary file 2 — Additional file 2:. Supplementary Materials and Methods. [file 12870_2021_3405_MOESM2_ESM.docx]

**New Insights into the Role of Cyanide in the Promotion of Seed Germination in Tomato**

**Lu-Lu Yu ^1^, Cui-Jiao Liu ^1^, Ye Peng ^1,2^, Zheng-Quan He ^2^, Fei Xu ^1,2*^**

^1^ Applied Biotechnology Center, Wuhan University of Bioengineering, Wuhan 430415, China;

^2^ Biotechnology Research Center, China Three Gorges University, Yichang 443002, China;

^*^ Correspondence: feixu501@whsw.edu.cn

**Supplementary Materials and Methods**

**1. Chemicals and reagents**

HPLC-grade acetonitrile (ACN) and methanol (MeOH) were purchased from Merck (Darmstadt, Germany). MilliQ water (Millipore, Bradford, USA) was used in all experiments. All of the stan-dards were purchased from Olchemim Ltd. (Olomouc, Czech Republic) and Sigma (St. Louis, MO, USA). Acetic acid was obtained from Sinopharm Chemical Reagent (Shanghai, China). The stock solutions of standards were prepared at the concentration of 10 mg/mL in ACN. All stock solutions were stored at -20 °C. The stock solutions were diluted with ACN to working solutions before analysis.

**2. Sample preparation and extraction**

For GA extraction and determination, the fresh plant materials were ground using a mixer mill (MM 400, Retsch, Germany) for 1 min at 30Hz. After that, samples were ground carefully and 0.2 g of the powdered sample was extracted overnight at 4°C with 1.5 mL 70% (V/V) acetonitrile. After vortex for 30 s and centrifugation at 14,000 rpm for 10 min, the supernatants (1.0 mL) were collected and then evaporated to dryness under nitrogen gas stream at room temperature, constituted in 100 μL 80% (V/V) methanol, diluted to 800 μl with water. The extracts were passed through the SPE cartridge (200 mg, 3 mL; CNW) and evaporated to dryness under nitrogen gas stream at room temperature. Following the sample were reconstituted in 200 μl 80% (V/V) methanol and filtrated (PTFE, 0.22 μm; Anpel) before LC–MS/MS analysis.

For ABA and IAA extraction and determination, the fresh plant materials were harvested, weighted, immediately frozen in liquid nitrogen, and stored at -80 °C until needed. 50 mg samples were ground with liquid nitrogen and extracted with 0.5 mL methanol/water/formic acid (15:4:4, V/V/V) at 4 °C. The extract was vortexed for 10 min and centrifuged at 14,000 rpm for 5 min at 4 °C. The supernatants were collected and repeated the steps above. The combined extracts were evaporated to dryness under nitrogen gas stream, reconstituted in 80% methanol (V/V), ultraphoniced (1 min) and filtrated (PTFE, 0.22 μm; Anpel) before LC-MS/MS analysis.

**3. HPLC conditions**

For GA determination, the sample extracts were analyzed using an LC-ESI-MS/MS system (HPLC, Shim-pack UFLC SHIMADZU CBM30A system, http://www.shimadzu.com.cn/; MS, Applied Biosystems 6500 Triple Quadrupole, http://www.appliedbiosystems.com.cn/). The analytical conditions were as follows, HPLC: column, Waters ACQUITY UPLC HSS T3 C18 (1.8µm, 2.1 mm*100 mm); solvent system, water (0.04% acetic acid): acetonitrile (0.04% acetic acid); gradient program, 80:5 (V/V) at 0 min, 50:95 (V/V) at 10 min, 35:95 (V/V) at 11 min, 25:5 (V/V) at 11.1min, 10:5 (V/V) at 14.0 min; flow rate, 0.35 mL/min; temperature, 45°C; injection volume: 5 μL. The effluent was alternatively connected to an ESI-triple quadrupole-linear ion trap (Q TRAP)-MS.

For ABA and IAA determination, the sample extracts were analyzed using an LC-ESI-MS/MS system (HPLC, Shim-pack UFLC SHIMADZU CBM30A system, www.shimadzu.com.cn/; MS, Applied Biosystems 6500 Triple Quadrupole, www.appliedbiosystems.com.cn/). The analytical conditions were as follows, HPLC: column, Waters ACQUITY UPLC HSS T3 C18 (1.8 µm, 2.1 mm*100 mm); solvent system, water (0.04% acetic acid): acetonitrile (0.04% acetic acid); gradient program, 90:10 (V/V) at 0 min, 40:60 (V/V) at 5 min, 40:60 (V/V) at 7 min, 90:10 (V/V) at 7 min, 90:10 V/V at 10 min; flow rate, 0.35 mL/min; temperature, 40°C; injection volume: 2 μL. The effluent was alternatively connected to an ESI-triple quadrupole-linear ion trap (Q TRAP)-MS.

**4. ESI-Q TRAP-MS/MS**

API 6500 Q TRAP LC/MS/MS System, equipped with an ESI Turbo Ion-Spray interface, operating in a positive ion mode and controlled by Analyst 1.6.3 software (AB Sciex). The ESI source operation parameters were as follows: ion source, turbo spray; source temperature 500°C; ion spray voltage (IS) 5500 V; curtain gas (CUR) were set at 35.0 psi; the collision gas (CAD) was medium. DP and CE for individual MRM transitions was done with further DP and CE optimization. A specific set of MRM transitions were monitored for each period according to the plant hormones eluted within this period.
